# Loss of Krüppel-like factor 9 deregulates both physiological gene expression and development

**DOI:** 10.1038/s41598-023-39453-3

**Published:** 2023-07-28

**Authors:** Laura Drepanos, Ian M. Gans, Janelle Grendler, Sophia Guitar, J. Heath Fuqua, Nathaniel J. Maki, Andrea R. Tilden, Joel H. Graber, James A. Coffman

**Affiliations:** 1 MDI Biological Laboratory, Salisbury Cove, ME USA; 2 Graduate School of Biomedical Sciences and Engineering, University of Maine, Orono, ME USA; 3 Colby College, Waterville, ME USA

**Keywords:** Computational biology and bioinformatics, Developmental biology, Molecular biology, Endocrinology

## Abstract

Krüppel-like factor 9 (Klf9) is a ubiquitously expressed transcription factor that is a feedforward regulator of multiple stress-responsive and endocrine signaling pathways. We previously described how loss of Klf9 function affects the transcriptome of zebrafish larvae sampled at a single time point 5 days post-fertilization (dpf). However, *klf9* expression oscillates diurnally, and the sampled time point corresponded to its expression nadir. To determine if the transcriptomic effects of the *klf9*^−/−^ mutation vary with time of day, we performed bulk RNA-seq on 5 dpf zebrafish embryos sampled at three timepoints encompassing the predawn peak and midmorning nadir of *klf9* expression. We found that while the major effects of the *klf9*^−/−^ mutation that we reported previously are robust to time of day, the mutation has additional effects that manifest only at the predawn time point. We used a published single-cell atlas of zebrafish development to associate the effects of the *klf9*^−/−^ mutation with different cell types and found that the mutation increased mRNA associated with digestive organs (liver, pancreas, and intestine) and decreased mRNA associated with differentiating neurons and blood. Measurements from confocally-imaged larvae suggest that overrepresentation of liver mRNA in *klf9*^−/−^ mutants is due to development of enlarged livers.

## Introduction

Krüppel-like factor 9 (Klf9), a ubiquitously expressed vertebrate member of the Krüppel-like family of transcription factors, has recently emerged as a critical regulator of multiple environmental- and stress-response pathways, including those mediated by the glucocorticoid receptor (GR), the thyroid receptor (TR), aryl hydrocarbon receptor (AHR), the redox homeostasis/oxidative stress factor Nrf2, and the proteostasis/ER-stress factor Xbp1^1–7^. Klf9 is also known to regulate both developmental cell differentiation and physiology in multiple tissue types and organ systems, including the central nervous system^8–11^, blood^2^, adipose tissue^12^, and liver^13^, and to interact with and regulate the circadian clock^14,15^. As is the case with many ubiquitously expressed transcription factors, the function of Klf9 depends on context, both spatially and temporally. While this limits broad generalizations about Klf9 function, evidence from various systems suggests that, like other KLFs, Klf9 often functions to regulate and/or fine-tune cellular responses to signal-responsive transcriptional activators, and that it often does so in incoherent feedforward loops, i.e., as a target whose activation functions to repress other targets^1,4,5,16,17^. Incoherent feedforward regulation is widely used in biological systems to optimize responsivity and mediate biochemical adaptation to signal strength^18–23^.

We previously showed that zebrafish *klf9* is transcriptionally upregulated by GR signaling and that the transcriptomic response to chronic cortisol treatment measured by bulk RNA-seq of zebrafish larvae is significantly altered by loss of *klf9* function^1,24,25^. We subsequently showed that *klf9* mRNA expression is oscillatory, with a diurnal rhythm that peaks just before dawn and then rapidly declines to a mid-morning nadir, a pattern also displayed by mRNA of the GR (and Klf9) target gene *fkbp5*, but not by several other GR target genes^17^. The diurnal oscillation of *klf9* transcript levels depends on a functional GR and is thus probably at least in part due to the diurnal oscillation in circulating cortisol levels^17^. Our previous RNA-seq experiment assessing the transcriptomic effects of the *klf9*^−/−^ mutation was carried out on pooled zebrafish larvae collected at a single mid-morning timepoint^1^, when *klf9* mRNA expression is at its nadir, leaving open the possibility that the observed effects were specific to that time of day.

Here we report the results of another bulk RNA-seq experiment that we carried out to assess the extent to which the transcriptomic effects of the *klf9*^−/−^ mutation depend on the time of day. As in our previous experiment, we sampled zebrafish larvae on day 5 post-fertilization, a full day after the hypothalamus-pituitary-interrenal (HPI, homologous to the mammalian hypothalamus–pituitary–adrenal or HPA) axis has developmentally matured and *klf9* expression has developmentally plateaued^17^. Samples were taken at three different time points: − 3, 0, and + 2.5 h with respect to the time when the lights come on (Zeitgeber Time, ZT), encompassing the *klf9* expression peak and nadir (respectively at ~ ZT0 and ZT+2.5). Four biological replicates each of wildtype (WT) and *klf9*^−/−^ larvae were sampled at each time point and subjected to bulk RNA-seq. We used the unbiased approach of Principal Component Analysis (PCA) to identify patterns of variance in the data, which were then mapped to the experimental design variables and other metadata to identify the sources of the variation. This analysis showed (unsurprisingly) that the transcriptome varies substantially with time of day, and that this dynamic is affected by the *klf9*^−/−^ mutation, such that a subset of the mutation’s effects manifests only at a specific time of day. Nevertheless, many effects of the mutation manifest independently of the time of day, including the major effects that we reported previously^1^. Using a published zebrafish larva single-cell transcriptome atlas^26^ we mapped the gene expression effects of the *klf9*^−/−^ mutation identified in our bulk RNA-seq experiments to putative cell and tissue type, which revealed potential functions of Klf9 in regulating the development of liver and other organ systems.

## Results

### Time of day, Klf9, and Zeitgeber contribute to expression variance among RNA-seq samples

**Figure 1:**
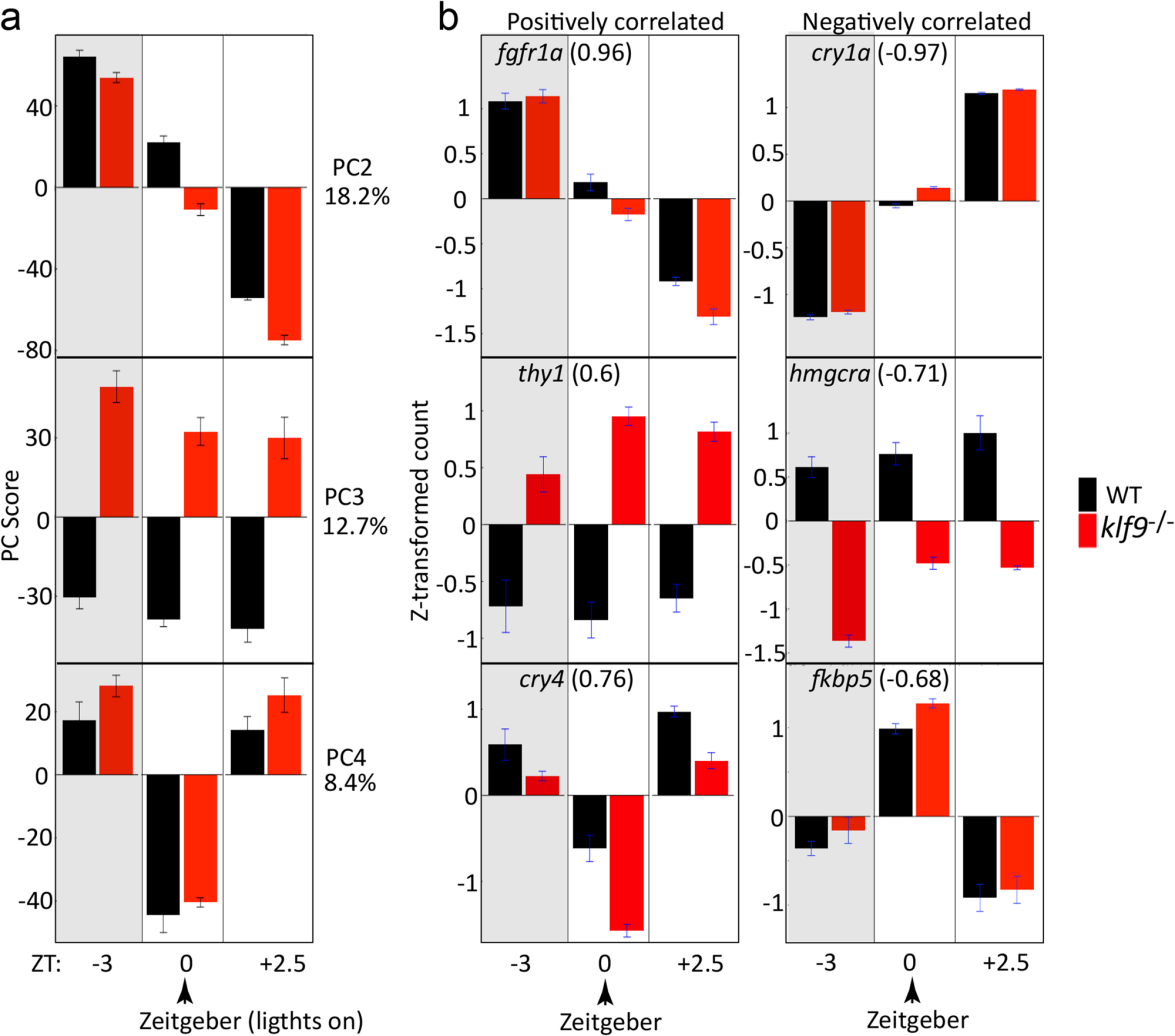
Principal Component Analysis reveals the effects of time of day, genotype, and Zeitgeber on variance between RNA-seq samples. (**a**) Average variance in expression values (y-axis) along PCs 2–4, with respect to time of day (x-axis) and genotype (red and black bars). Error bars are the SEM for the four biological replicates. PC1 was determined to be a likely technical artifact (Supplemental Figs. S1–3). (**b**) Example genes whose expression variance was positively or negatively correlated with PCs 2–4. The Pearson correlation coefficient of the gene’s expression variance with the PC is shown in parentheses.

Principal component analysis (PCA) of the RNA-seq data was performed to identify patterns of variance in the transcriptomes among four biological replicate samples taken from two genotypes (wildtype and *klf9*^−/−^) at each of three time points (ZT−3, ZT0, and ZT+2.5) (Fig. 1a, Supplementary Fig. S1). Surprisingly, the first principal component, which captured 26.86% of the variance, indicated systematic differences between biological replicate samples within each experimental treatment group (time and genotype) (Supplementary Fig. S2). This was revealed in follow-up experiments to be in large part, and possibly entirely, a technical artifact of the procedure used for mRNA extraction and purification (see Methods, Supplementary Fig. S3). PC1 was therefore used as a covariate in the differential expression analyses described below to identify the effects of Klf9 loss. PC2 captures 18.24% of the variance in gene expression and revealed a monotonic variation with the time of day that the samples are collected. Genes whose expression variance correlated with this PC (Supplementary Table S4) included *fgf1ra* (positively correlated) and the circadian regulator *cry1a* (negatively correlated) (Fig. 1b, Supplementary Fig. S4). Notably, compared to wildtype, *klf9*^−/−^ variance along PC2 was consistently displaced toward the negative pole, an effect that is most pronounced at ZT0. PC3 captures 12.73% of the variance and correlates with the presence or absence of Klf9 (Fig. 1a). Genes whose expression variance correlated with this PC (Supplementary Table S4) included *thy1* (positively correlated) and *hmgrca* (negatively correlated) (Fig. 1b, Supplementary Fig. S4). PC4 captures 8.4% of the variance and reveals a distinct expression pattern that segregates samples collected at ZT0 (onset of light in the incubator) from those collected at ZT−3 and ZT+2.5. Genes whose expression variance correlated with this PC (Supplementary Table S4) included *cry4* (positively correlated) and *fkbp5* (negatively correlated) (Fig. 1b, Supplementary Fig. S4).

### Loss of Klf9 function has both time-of-day independent and time-of-day dependent effects on gene expression

**Figure 2:**
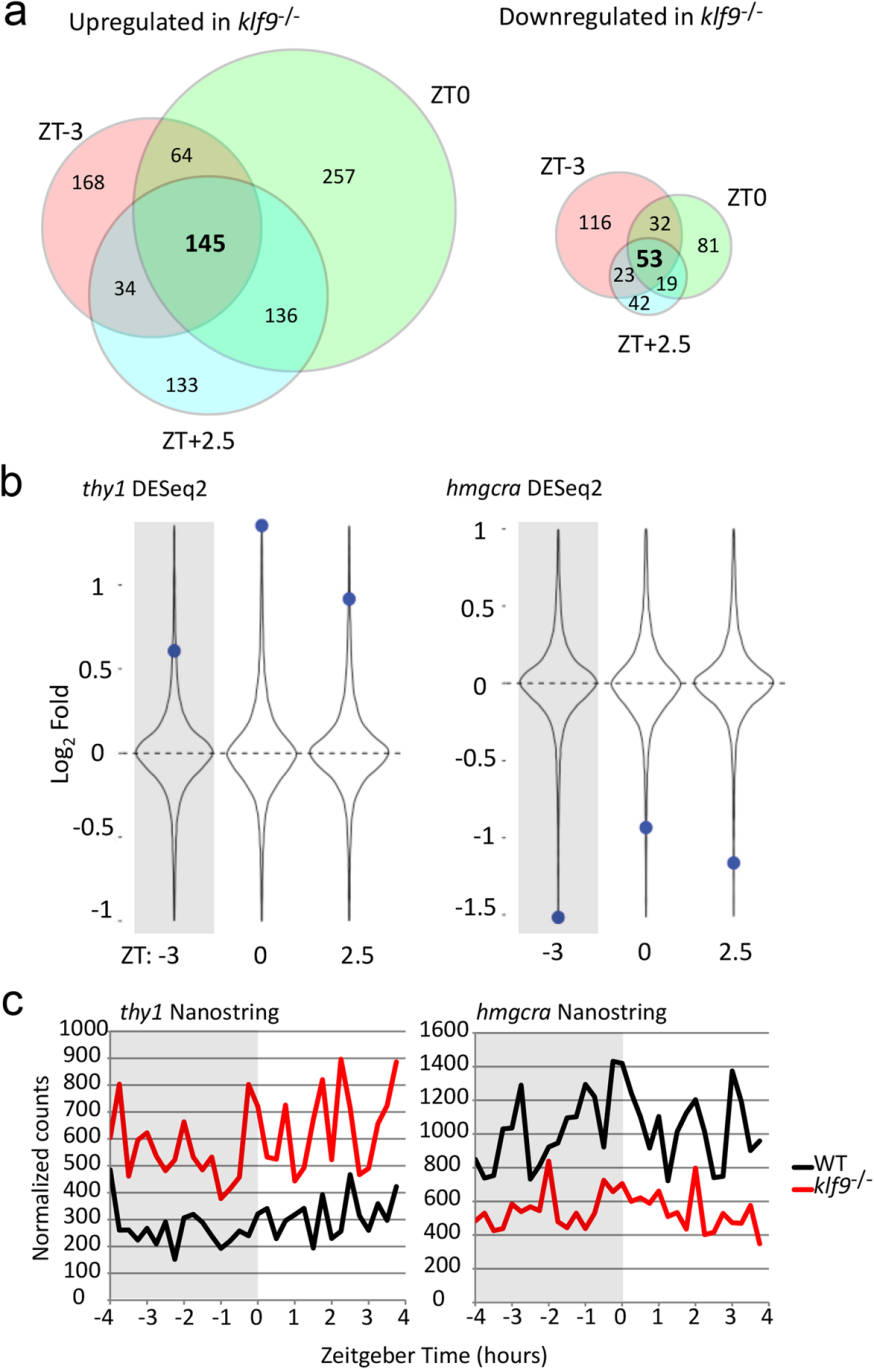
Loss of Klf9 function affects expression of some genes irrespective of time of day. (**a**) Venn diagrams (produced using Venny;^54^) showing numbers of genes found by DESeq2 to be differentially expressed (log_2_FC ≥ 0.5, FDR ≤ 0.1) between *klf9*^*−/−*^ and wildtype larvae at each time point. (**b**) *klf9*^*−/−*^ induced differential expression of *thy1* and *hmgcra* with respect to DESeq2 distribution at each time point. In this and all following figures, the violin plot shows the distribution of values across all measured genes. (**c**) Nanostring analysis of *thy1* and *hmgcra* expression with respect to time over an 8-h time course. Each sample consisted of total RNA pooled from 4–5 larvae collected at the indicated times on 5 dpf (see Gans et al., 2021, ref.^17^).

Differential gene expression (DESeq2) analysis was performed to specifically assess how loss of Klf9 function affects gene expression, comparing wildtype and *klf9*^−/−^ mutant samples both for the overall experiment (all time points combined) and independently at each time point (Supplementary Table S5). This analysis confirms that loss of Klf9 function affects a subset of genes irrespective of time of day (as also indicated by PC3, Fig. 1a) and suggests as well that some genes are affected by the mutation only at specific times of day (Fig. 2a; Supplementary Table S6). Interestingly, many more genes were found to be upregulated than downregulated in the *klf9*^−/−^ mutants (937 vs. 366), reinforcing previous findings that suggested that Klf9 functions more often as a repressor than as an activator of transcription^27,28^.

Gene-ontology term enrichment analysis of gene lists ranked according to either their position on PC3 (Supplementary Table S4) or their ranking by DESeq2 fold-change (Supplementary Table S5) across all time points indicates that Klf9 is critical for regulating genes involved in various aspects of metabolism and the immune system (Supplementary Figs. S5 and S6), consistent with our previous reports^1,17^. Our previous study^1^ found that, compared to wildtype controls, *klf9*^−/−^ mutants overexpress genes associated with the complement system, an effect that was reproduced here, although only a subset of complement genes are overexpressed (Supplementary Fig. S7A, Table S7). We also previously reported that genes associated with sterol biosynthesis (e.g., *hmgcra*) were downregulated in *klf9*^−/−^ mutants, which as noted above was also reproduced here. However, DESeq2 analysis of all genes associated with sterol biosynthesis showed that some are overexpressed in the mutants, indicating that the mutation has complex effects on that biological process (Supplementary Fig. S7B, Table S7). Some of these effects are further elucidated in the single cell mapping described below in the next section.

The PCA described above showed that two genes among those most strongly affected by loss of Klf9 function irrespective of time of day were *thy1*, which encodes a widely expressed cell surface protein that mediates cell–cell and cell–matrix signaling interactions that negotiate developmental processes^29^, and *hmgcra*, which encodes the rate-limiting enzyme in cholesterol synthesis^30^ (Fig. 1b)*.* This was confirmed by our DESeq2 analysis (Fig. 2b), and independently by Nanostring analysis of a high-density time course experiment that we reported previously (Fig. 2c).

**Figure 3:**
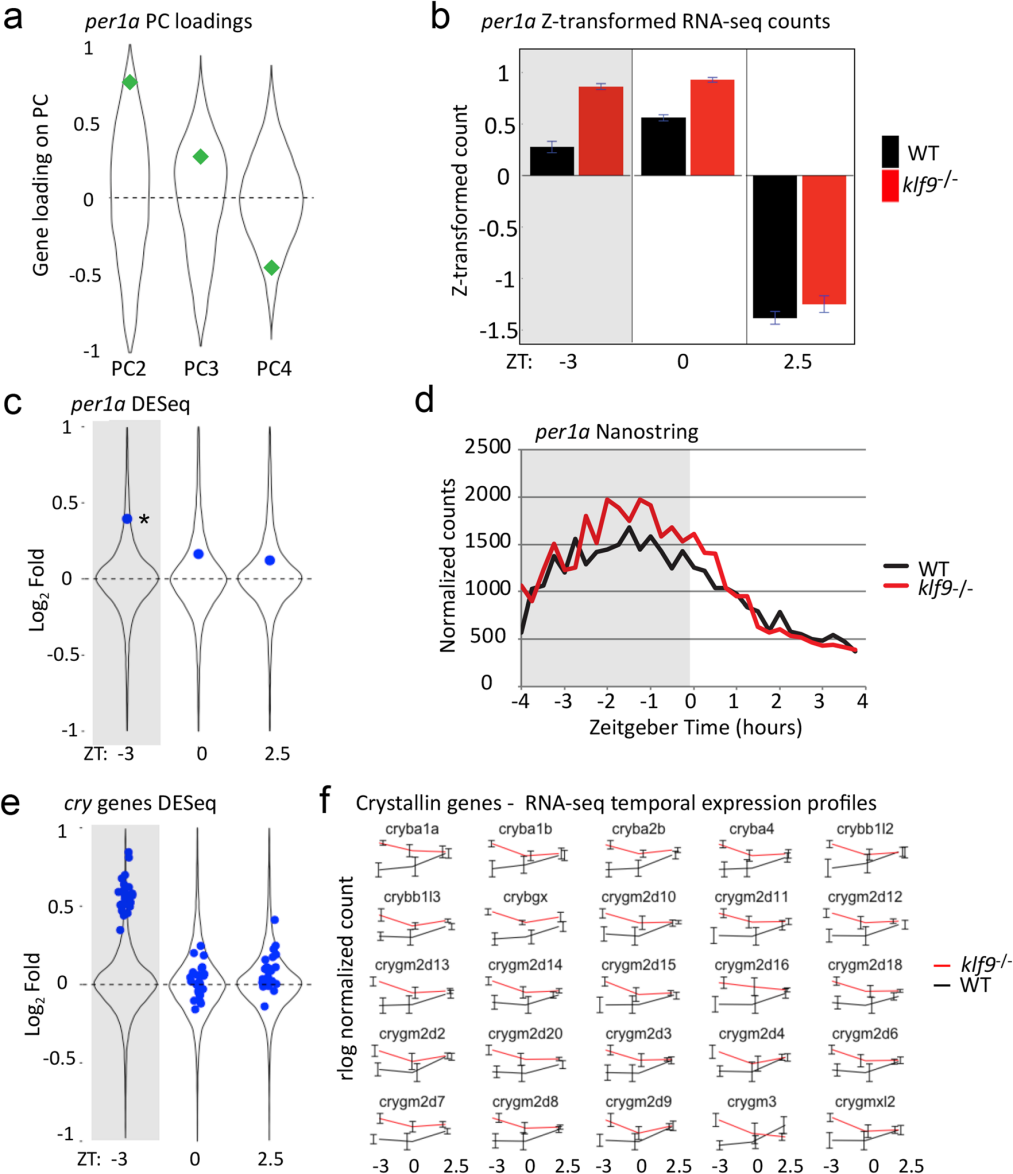
Loss of Klf9 function affects expression of *per1a* and *crystallin* genes only at a specific time of day. (**a**) Correlation of *per1a* expression variance with PCs 2–4. (**b**) Mean normalized expression of *per1a* at each time point in each genotype; error bars indicate the SEM. (**c**) *klf9*^*−/−*^ induced differential expression of *per1a* with respect to the DESeq2 distribution at each time point; *Adjusted *p*-value = 0.0013. (**d**) Nanostring analysis of *per1a* expression with respect to time over an 8-h time course. Each sample consisted of total RNA pooled from 4–5 larvae collected at the indicated times on 5 dpf (see Gans et al., 2021, ref.^17^). (**e**) *klf9*^*−/−*^-induced differential expression of 25 *cry* genes with respect to the distribution of all genes that are differentially expressed between *klf9*^*−/−*^ and WT at each time point. (**f**) Temporal profiles of *cry* gene expression. Error bars are the SEM of the four biological replicates.

Many genes were affected by more than one of the experimental variables, as shown by their loadings on different PCs. An illustrative example is the circadian regulator *per1a*, whose expression variance was highly correlated with PC2 (as expected), but which also showed modest correlation with PCs 3 and 4 (Fig. 3a). While the PCA suggested that the *klf9*^−/−^ mutation affects *per1a* expression at all three time points (Fig. 3b), a significant effect was detected by DESeq2 only at ZT-3 (Fig. 3c), although the magnitude of the effect was relatively small (Log2 Fold Change of 0.39). Nevertheless, independent measurements using the Nanostring platform reproduced the effect (Fig. 3d). Thus, although *per1a* was not identified in the DESeq2 results shown in Fig. 2a (owing the stringency of the fold-change and false discovery rate thresholds), the data depicted in Fig. 3, together with the fact that the mammalian homolog *Per1* is known to be a direct regulatory target of Klf9^31^, suggest that its predawn expression is negatively regulated by Klf9. A similar time-of-day dependent effect was observed for some other circadian genes, including *dbpb*, whose mammalian homolog *Dbp* is a known Klf9 target^14^, and *nr1d2a*, whereas core clock genes such as *clocka*, *clockb*, *per2*, and *per3* were unaffected by the *klf9*^−/−^ mutation (Supplementary Table S5). Consistent with loss of Klf9 having time-of-day specific effects, gene ontology term enrichment analysis of the DESeq2 comparing wildtype and *klf9*^−/−^ larvae at each of the three time points yielded distinct biological processes (Supplementary Figs. S8–S10).

The DESeq2 analysis also revealed that genes encoding Crystallin proteins involved in the formation of the optic lens are differentially expressed in *klf9*^−/−^ mutants at only one of the three time points (ZT−3, Fig. 3e). In wildtype zebrafish, expression of those genes is low at ZT−3 and ZT0, then increases by ZT+2.5 (Fig. 3f). In contrast, in *klf9*^−/−^ mutants the genes are most highly expressed at ZT−3, after which their expression declines, converging on wildtype levels at ZT0 and ZT+2.5 (Fig. 3f, Supplementary Fig. S11).

### Klf9-dependent gene expression is associated with specific cell types

**Figure 4:**
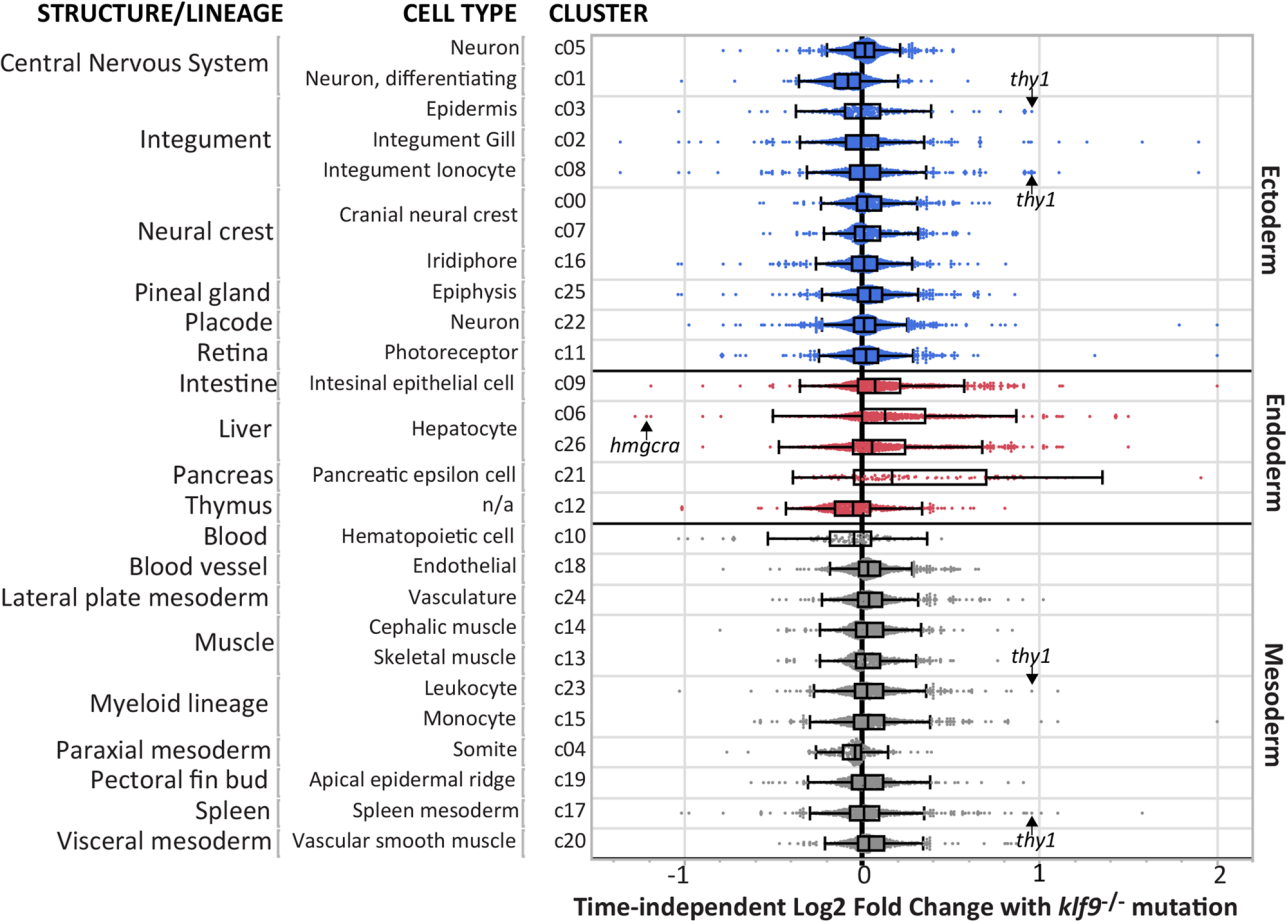
Effect of the *klf9*^*−/−*^ mutation mapped onto different cell types identified in the single cell atlas of zebrafish development. Differentially expressed genes were mapped to cell clusters identified in the atlas of Farnsworth et al. (ref.^26^) and annotated as described in the text (see Supplemental Materials, Table S3). Each differentially expressed gene is represented as a dot within the cluster. The locations of *hmgcra* (downregulated in *klf9*^*−/−*^ mutants) and *thy1* (upregulated in *klf9*^*−/−*^ mutants) are shown.

To associate the effects of the *klf9*^−/−^ mutation with specific cell and tissue types we used the single-cell atlas of zebrafish development published by Farnsworth et al.^26^, as described in the Methods. Assuming that loss of Klf9 does not produce significant ectopic expression of genes used to identify cell types (an assumption that remains to be tested, e.g., by in situ hybridization), two single-cell clusters annotated as liver are significantly overrepresented in the mutants, as are other endodermal derivatives involved in digestion (intestine and pancreas; Fig. 4). In contrast, clusters representing differentiating neurons, thymus, and hematopoietic cells are underrepresented in the mutants (Fig. 4). As might be expected, increased expression of complement cascade genes in *klf9*^*−/−*^ mutants^1^ maps to liver cells (Supplementary Table S3). In addition to identifying specific clusters that were overall either over- or under-represented in *klf9*^*−*/*−*^ mutants compared to wildtype, this analysis indicated that numerous genes within each cluster (including clusters whose average representation did not change) were differentially expressed in the mutants. Examples are *hmgcra*, which as noted above is significantly downregulated in *klf9*^*−*/*−*^ mutants but specifically associated with the liver (hepatocyte) cluster that overall is overrepresented, and *thy1*, which is significantly upregulated in *klf9*^*−*/*−*^ mutants but associated with four clusters (epidermis, integument ionocyte, leukocyte, and spleen mesoderm) that do not change in overall representation (Fig. 4).

### Klf9^−/−^ mutants develop enlarged livers

**Figure 5:**
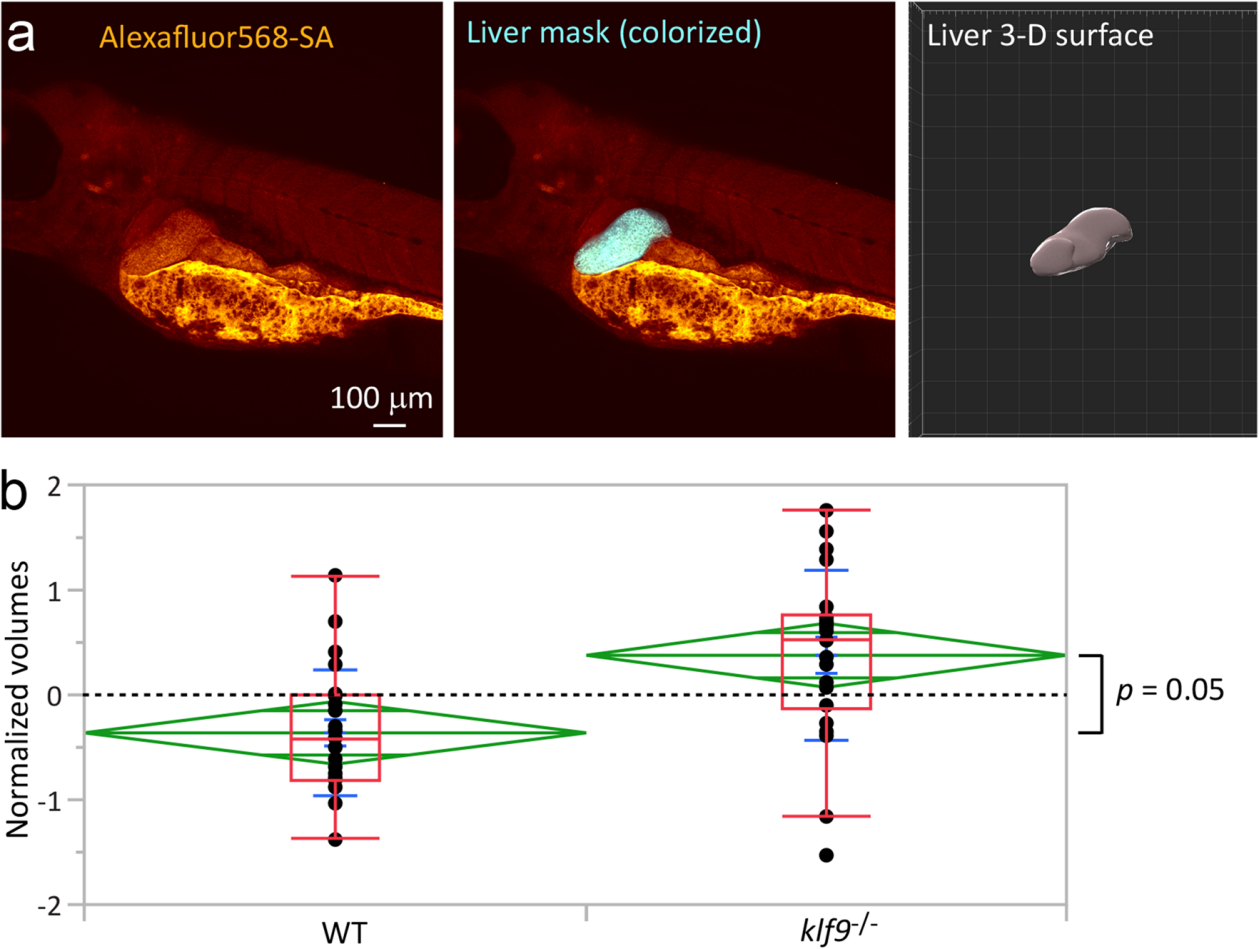
Klf9^*−/−*^ mutants have larger livers than wildtype larvae. (**a**) Image of Alexafluor568-Streptavadin-stained larva showing how the liver was located and segmented to create a 3-D surface for estimating its volume in Imaris. (**b**) Measurements of liver volumes, combined from separate analyses performed blind by two different individuals. The two sets of measurements differed slightly but systematically, so were brought into register by mean-centering all measurements taken by each experimenter before integrating their data (see Methods and Supplemental Fig. S8). This allowed each individual measurement to be plotted as a quantity relative to the average (“0” on the y-axis). The green diamonds show the 95% confidence interval of the mean, which is indicated by the central horizontal green line; the other two horizontal green lines delimit the region within which the two means are not significantly different. Significance was calculated by t-test.

The observation that *klf9*^*−*/*−*^ mutants overexpress many genes associated with liver suggests that the mutants may have more total RNA within liver cells (e.g., via hepatocyte hypertrophy), or that they have more liver cells, or both. Either scenario would be expected to manifest as enlarged livers in *klf9*^*−*/*−*^ mutants compared to wildtype controls. To determine how the *klf9*^*−*/*−*^ mutant affects liver size we stained fixed 5 dpf larvae with fluorescent streptavidin, which labels liver, intestinal epithelium, and yolk^32^, and imaged the stained larvae confocally. Since the livers can be distinguished morphologically from the other stained structures, this allowed us to estimate liver volumes in images of the liver surface reconstructed from confocal z-stacks (Fig. 5a). The *klf9*^*−*/*−*^ mutants were found to have significantly larger livers than WT (Fig. 5b and Supplementary Fig. S12), confirming that the increased liver gene expression observed in the mutants is at least in part due to increased number and/or size of liver cells (hepatocytes and/or Kupffer cells). Further work (e.g., using DAPI staining of nuclei) is needed to determine whether hypertrophy or hyperplasia (or both) account for the liver overgrowth in *klf9*^*−*/*−*^ mutants.

## Discussion

RNA-seq is commonly used to comprehensively assess changing patterns in gene activity associated with development and/or physiology under different genetic and/or environmental conditions. Originally the technique was applied to whole tissues, organs, or organisms (“bulk” RNA-seq), but more recently single-cell RNA-seq (scRNA-seq) methods have allowed investigators to assess effects of experimental variables on specific cells and thus the overall effect on different cell populations. A limitation of scRNA-seq compared to bulk RNA-seq is that the depth of sequencing is much lower, and thus less sensitive to subtle transcriptomic effects. Recently methods have been developed that allow bulk RNA-seq results to be mapped to cell types resolved in scRNA-seq atlases^33–35^, increasing the cell/tissue-specific resolution of the bulk RNA-seq. The analyses described here interrogated bulk RNA-seq data to infer functions of the ubiquitously expressed, stress-responsive transcription factor Klf9 in zebrafish, via two objectives: (1) given our recent finding that *klf9* expression oscillates diurnally^17^, we sought to determine the extent to which the transcriptomic effects observed in our *klf9*^*−*/*−*^ mutant depend on the time of day the samples were obtained; and (2) given the availability of a recently published single cell atlas of zebrafish development^26^, we sought to map the transcriptomic effects of the *klf9*^*−*/*−*^ mutation to specific cell types.

We began by using Principal Component (PC) analysis to parse the variance in the bulk RNA-seq data irrespective of the experimental design variables, an unbiased approach that allows the variance to be correlated with those variables and other experimental metadata post hoc. Based on previous results (unpublished, manuscript in preparation) indicating that the acute stress of sample collection can introduce a stress-response gene expression signature, we took great care in this experiment to eliminate acute stress as a variable between serially collected biological replicates (see "Methods"). Despite this, we found that the largest source of variance in the data (PC1) was between biological replicates *within* each experimental condition (Supplementary Fig. S2). In follow-up qRT-PCR and Nanostring experiments designed to determine whether inter-replicate variance was biological and/or reproducible, we found that while it was replicated by the column purification method that was used for the RNA-seq, it was largely eliminated when the RNA was purified using the Trizol method (Supplementary Fig. S3 and data not shown). Subsequent experiments revealed that the variance was introduced by the variable time that each sample was incubated on ice in RLT+ buffer, which we had not temporally controlled (unlike the incubation in Trizol). We found that when the incubation time in RLT+ buffer was controlled prior to column purification, the variance decreased (data not shown). We conclude that the experimental protocol and technique used to prepare the RNA for sequencing requires careful attention to detail, as does the recording of metadata associated therewith, as variables that may seem innocuous can introduce significant systematic variance between replicate samples. Despite this unforeseen technical variation, we were able to subtract the noise that it introduced by using PC1 as a covariate in the subsequent analyses (see "Methods"). The next three largest sources of variance (captured in PCs 2–4) were biological, correlating with time of day, genotype (*klf9*^−/−^ mutant vs. wildtype), and Zeitgeber (the onset of the light phase in the incubator) (Fig. 1). As we were specifically interested in understanding Klf9 function, we focused mainly on PC3, representing variance in the gene expression driven by the *klf9*^−/−^ mutation. It is important to note however that the *klf9*^−/−^ mutation also affected the variance captured in PCs 2 and 4 (time of day and Zeitgeber, respectively), as shown by the consistent differences in *klf9*^−/−^ and WT seen along those PCs (Fig. 1). This suggests that genes whose expression varies with time of day and/or responds to the Zeitgeber are also affected by the *klf9* null mutation, albeit to a smaller degree.

Two genes that segregated strongly to opposite poles of PC3 were *thy1* and *hmgcra* (Fig. 2). Thy1 (CD90), a cell surface protein expressed in multiple cell types, functions as a signaling scaffold to negotiate microenvironmental cell–cell and cell–matrix interactions during development^29^. HMGCR (HMG-CoA Reductase) is the rate-limiting enzyme in cholesterol biosynthesis^30^. While the biological significance of *thy1* upregulation and *hmgcra* downregulation in *klf9*^−/−^ mutants is not clear, we can surmise that it may relate to Klf9 function in regulating development. In various contexts, Klf9 has been shown to be important for terminal cell differentiation and concomitant cessation of cell proliferation, a role that is consistent with studies that indicate that it often functions as a tumor suppressor^28,36–38^. Thus, one might expect *klf9*^−/−^ mutants to show signs of underdevelopment such as impeded cell differentiation, and perhaps also loss of growth regulation. Indeed, the results of the single cell integration analysis (Fig. 4) provide indications of both, albeit in different tissues: impeded differentiation in *klf9*^-/-^ mutants is suggested by lower representation of genes associated with ectodermal and mesodermal derivatives dedicated to environmental interaction (differentiating neurons and blood), whereas loss of growth regulation is suggested by higher representation of genes associated with endodermal derivatives dedicated to metabolism (liver, intestinal epithelium, pancreas) (Fig. 4). The observed underrepresentation of hematopoietic cells in *klf9*^-/-^ mutants is consistent with a previous report showing that Klf9 promotes hematopoiesis during zebrafish development^2^. Moreover, the overexpression of *thy1*, a marker of lineage immaturity^39,40^ associated with multiple cell types (Fig. 4) is consistent with impeded lineage maturation. Altogether then, it is reasonable to hypothesize that gene expression effects that manifest independent of time of day are at least in part reflective of impeded development in the mutants. Further analyses are ongoing to characterize the developmental effects of the *klf9*^−/−^ mutation and will be the subject of future reports.

In the case of the liver, the hypothesis that the increased representation in *klf9*^−/−^ mutants of genes associated with that organ is due to its overgrowth is supported by measurements obtained of the volumes of fluorescently stained and confocally imaged livers (Fig. 5). While Klf9 has long been known to play important roles in liver physiology and pathogenesis^3,13,38^, to our knowledge this is the first report of it playing a role in regulation of liver growth during development. In future work it will be important to determine whether overgrowth of livers in *klf9*^−/−^ mutants is due to hepatocyte hyperplasia, hypertrophy, or both. In this regard it is interesting that *hmgcra* is significantly downregulated in *klf9*^−/−^ mutants, as it encodes a liver enzyme and is specifically associated with hepatocytes in the single cell analysis. This suggests that even though *klf9*^−/−^ develop enlarged livers, the hepatocytes are physiologically abnormal, which given the discussion above may reflect immaturity. Since many of the genes regulated by Klf9 encode regulators of energy metabolism and redox homeostasis^4,12,13,17^, it is tempting to speculate that some of the developmental effects of the *klf9*^−/−^ mutation are indirect consequences of altered metabolism, which resonates with the emerging appreciation of the old idea that metabolism plays critical roles in directing development, often via redox signaling^41,42^.

In addition to the time-of-day independent effects, our analysis revealed effects of the *klf9*^−/−^ mutation that were only apparent at a specific time of day. The affected genes included the circadian regulator *per1a*, the mammalian homolog of which is a known Klf9 (and GR) target^31^. The fact that *per1a* is overexpressed in *klf9*^−/−^ mutants only at the predawn timepoint may simply reflect that that is its normal expression peak, which coincides with that of *klf9*^17^. Notably however, *klf9*^−/−^ mutants also overexpress a battery of genes encoding Crystallin proteins (*cry* genes) at ZT−3 but not ZT0 and ZT+2.5 (Fig. 3). In wild-type larvae *cry* gene expression is low at the pre-dawn time points, then increases after the lights come on, whereas the opposite dynamic is observed for these genes in *klf9*^−/−^ mutants (Fig. 3b). In rodents, *Cry* gene expression is regulated by both light and the circadian clock^43^. Given that Klf9 often functions as a repressor and has been implicated in circadian regulation^14,15,27,28,44^, our data suggest that it may function as a repressor to enforce lower nocturnal *cry* gene activity. Further work examining the *cis*-regulatory systems controlling *cry* gene expression is needed to test this. Altogether, the observation that loss of *klf9* function affects the expression of these genes at a specific time of day corresponding to peak *klf9* expression is consistent with a physiological role for Klf9 in controlling transcriptional responsivity to cues associated with biological rhythms generated by the internal circadian clock and/or external Zeitgebers. A physiological (as opposed to developmental) role for Klf9 is also suggested by the fact that many genes were either over- or under-expressed in specific cell or tissue types that were not on the whole over- or under-represented in the *klf9*^−/−^ mutants (Fig. 5).

Interestingly, a transcriptomic response to the Zeitgeber is captured in PC4 of our analysis (Fig. 1). It is not unexpected that the transcriptome would respond in a concerted way to the onset of light (or any other Zeitgeber). That the response is indeed to the onset of light is suggested by the strong correlation of PC4 with the expression of *cry4* (Fig. 1b), which encodes a light-responsive cryptochrome protein^45,46^. Surprisingly, a gene whose expression variance is negatively correlated with PC4 is *fkbp5* (Fig. 1b), a well-known target and negative feedback regulator of the GR that we recently showed to also be negatively regulated by Klf9^17^. Notably, the analysis reported here did not detect a significant effect of the *klf9*^−/−^ mutation on *fkbp5* expression, although the positive shift in *fkbp5* position along PC4 in the mutants (Fig. 1b) is consistent with our previous results. We interpret this apparent discrepancy as reflecting the lack of temporal resolution in the present study compared to that of our previous analysis, which used high-density sampling (every 15 min) over an 8-h time course (as shown here for *thy1*, *hmgcra*, and *per1a* in Figs. 2 and 3). In any case, the fact that variance in *fkbp5* expression is most strongly (negatively) correlated with PC4 suggests that its temporal dynamic is entrained by the Zeitgeber. Given our previous results^17^, Klf9 may contribute to this regulation. The observation that many more genes are upregulated at ZT0 in Klf9 mutants than at the other two time points (Fig. 2a) is also suggestive of a role for Klf9 in regulating the transcriptomic response to the Zeitgeber. Finally, it is worth pointing out that the transcriptomic response captured in PC4, which was not included as a variable to be queried in the experimental design, might have been easily overlooked a priori and missed in an analysis that did not take the unbiased approach of PCA. In that case the variance would have been treated as noise rather than signal attributable to a biological stimulus–response relationship. This again highlights the critical importance of attention to detail in recording experimental metadata, as doing so allows observed patterns of variance to be attributed to experimental variables that are not designed or that cannot be controlled.

In summary, as has been recently shown by other groups using different approaches^33–35^, the analyses described here indicate that bulk RNA-seq can be used to infer tissue-specific developmental and/or physiological effects by taking advantage of existing single-cell atlases to resolve the differential gene expression patterns into source cell and tissue type, generating testable hypotheses to account for the observed pattern that can then be tested using other techniques such as microscopic imaging, as we have done here. This allows us to take advantage of both the higher sensitivity bulk RNA-seq provides for detecting subtle effects, as well as the economic advantages it provides for sampling over time and with multiple experimental variables. We have also shown the power of Principal Component analysis for detecting variance in the data that may or may not be associated with the experimental design variables, increasing the sensitivity of the analysis compared to conventional approaches that only do pairwise comparisons to assess differential gene expression without accounting for all sources of variance. Our analyses provide further evidence that the transcription factor Klf9 contributes globally to the regulation of both development and physiology, with a critical role controlling liver development, consistent with previous studies showing that Klf9 function is important for the regulation of metabolism^11–13,17^.

### Limitations of the study

Although the results depicted in Fig. 4 suggest that endodermal cell types (intestinal epithelium, hepatocyte, and pancreatic epsilon cells) are overrepresented in *klf9*^−/−^ mutants (based on the differential expression of cell-type specific marker genes used to assign identities to cell clusters in the single cell atlas), we cannot exclude the formal possibility that the overrepresentation of those genes in *klf9*^−/−^ mutants is due to their ectopic expression in cell types that normally don’t express them. Further experiments using in situ hybridization are required to test that possibility. Nevertheless, the results depicted in Fig. 5 indicate that at least in the case of liver the interpretation that hepatocytes are overrepresented in *klf9*^−/−^ mutants is correct. A second limitation is that the measurements depicted in Fig. 5 do not allow us to determine whether the increase in liver size is due to hepatocyte hyperplasia or hypertrophy (or both). As noted above, further work, e.g., involving quantitative fluorescence microscopy of livers with stained nuclei, is required to answer that question.

## Methods

### Zebrafish husbandry, culture, and collection

Zebrafish were *klf9*^−/−^ mutants derived from the AB strain^1^, and wildtype controls derived from the same grandparents. Husbandry and procedures were as described previously^25^. All animal procedures were approved by the Institutional Animal Care and Use Committee (IACUC) of the MDI Biological Laboratory, and all methods were performed in accordance with the relevant guidelines and regulations**.** The study is reported in accordance with ARRIVE guidelines.

Embryo culture and cortisol treatments were performed as previously described^1^. Briefly, fertilized eggs were collected in the morning, disinfected, and at ~ 4 h post fertilization placed in dishes with embryo media. Embryos developed in a 28.5 °C incubator with a 14/10 light/dark cycle synchronized with the core fish room. Media was changed daily.

### RNA extraction and purification

At − 3, 0, and + 2.5 h zeitgeber time (onset of the light phase in the incubator) on day 5 post-fertilization, four biological replicates of *klf9*^−/−^ mutant and wildtype larvae were collected as follows. Each biological replicate from each genotype and timepoint consisted of six larvae in a 30 mm dish in 4 ml of media. Larvae were collected from the dish into Eppendorf tubes, media was removed, and larvae immediately snap frozen in liquid nitrogen. Collection of the 8 samples at each time point occurred over ~ 10 min. Frozen samples were stored at − 80° C. RNA was prepared using the Qiagen RNeasy Plus mini kit (Qiagen 74134). For each set of four biological replicate samples, RLT Plus lysis buffer (plus beta-mercaptoethanol, per kit instructions) was added to each sample immediately upon its retrieval from the freezer, and the sample was then immediately homogenized with a motorized pestle and placed on ice before the next sample was retrieved from the freezer and subjected to the same procedure. Following pestle homogenization replicate samples received simultaneous processing—i.e., passed through Qiashredder columns (Qiagen 79654), bound, washed, and eluted per kit instructions. RNA was quantified and stored at – 80 °C.

For follow-up experiments using quantitative reverse transcription and polymerase chain reaction (qRT-PCR) and Nanostring, RNA was prepared either by the column method as described above or by the Trizol method, and for Nanostring analysis RNA was prepared by the Trizol method. The Trizol method includes a controlled incubation time of 5 min in Trizol immediately after homogenization. This step was not included in the RNeasy plus method described above, which had variable times in the homogenization buffer (longest for the first sample retrieved from the freezer, shortest for the last sample). We therefore tested whether this variable might be a source of variance in the data by controlling the incubation time in RLT+ buffer at 5 min per sample and found that this significantly reduced the variance between samples.

### RNA-seq and data analysis

Frozen RNA was sent to the Oklahoma State Genomics Facility for Illumina library preparation and sequencing. RNA-seq libraries were generated with Illumina-compatible KAPA libraries and sequenced as single-end 75 bp reads on an Illumina NextSeq 500 High Output sequencer. Quality and adapter trimming was applied to fastq formatted read files with Trim Galore Version 0.6.5^47^ with options: “--fastqc_args ‘--noextract’”, “--illumina”, “--length_1 35”, “--length_2 35”, “--stringency 1”, “--length 20”, and “--quality 20”. Gene and isoform expression were estimated with RSEM Version 1.3.3^48^ using Ensembl release 101^49^ of the *Danio rerio* genome for alignment and using command “rsem-calculate-expression” with options: “--keep-intermediate-files”, “--star-gzipped-read-file”, “-p 64”, “--estimate-rspd”, “--append-names”, “--output-genome-bam”, and “--star”. RSEM estimates of sample-specific expression were merged using R (version 4.0.3) package tximport version 1.0.3^50^.

For downstream analysis, it is more valuable to refer to genes by their name to estimate expression. There were 581 genes that were associated with more than one Ensembl ID, so the count matrix collapsed these distinct transcript counts by taking the sum of all transcript counts that correspond to the same gene symbol (retaining the earliest EnsemblID), effectively removing gene duplicates from the count matrix.

Differential Expression Analysis was performed between wildtype and *klf9*^−/−^ samples in the count matrix with DESeq2, version 1.30.1^51^, using R version 4.0.3. DESeq2 was also used to generate a rlog-matrix which was subsequently Z-transformed (subtracting gene average over all samples and then dividing by the gene’s standard deviation) to normalize each gene across all samples. The Z-transformed rlog matrix was then loaded into JMP version 16 and used for PCA (using the “Wide Method”). As described below, the first PC in all analyses was attributed to a technical effect, therefore the PC coordinate was subsequently used as a covariate in DESeq2 differential expression analysis. Four DESeq2 runs were performed: *klf9*^−/−^ vs WT samples at each of the three time points, and then overall with time as a covariate to determine the time-independent effect of *klf9*. Gene Ontology Term Enrichment analysis was performed using GOrilla^52^, and only included genes in the top 90% of baseMean counts and bottom 85% of logfoldchangeSE. Gene ontology treemaps of the GOrilla results were generated using REViGO^53^.

### Nanostring and qRT-PCR analysis

Nanostring analysis was performed at the Dartmouth Molecular Biology Core Facility on samples collected over an 8-h time course that we described previously^17^, using custom PlexSet probes designed by Nanostring (Supplemental Materials, Table S1). For qRT-PCR, cDNA was synthesized using the Primescript reverse transcription kit (Takara RR037a). Real time PCR was performed on a Roche LightCycler II thermal cycler using the Perfecta SYBR Green Fastmix (VWR 101414-270) and primers for several genes associated with the two poles of PC1 of the RNA-seq (Supplemental Materials, Table S2).

### Mapping differentially expressed genes to specific tissues and cell types

To map the effects of the *klf9*^−/−^ mutation onto cell and tissue type we took advantage of a recently published single-cell atlas of zebrafish development^26^. For the purposes of this analysis, we reduced the single-cell data to just those obtained at 5 dpf and used Seurat to identify clusters and marker genes for those clusters. Probable cell types within the clusters were identified by matching the annotations from the full embryo atlas to the 5 dpf single-cell data. Our time-independent bulk RNA-seq differential gene expression results were then reduced to only genes identified as marker genes in the single-cell data (Supplemental Materials, Table S3).

### Fixation, staining, and mounting of zebrafish larvae for imaging

Staining and imaging of livers was carried out following the protocol of Sadler et al.^32^. Briefly, 5 dpf larvae were fixed overnight in 4% formaldehyde at 4 °C, washed 3 × with PBS + 0.1% Tween-20 (PBST), then dehydrated through a methanol/PBST series (20/80 → 50/50 → 80/20 → 100% methanol), and stored in methanol at 4 °C. Fixed larvae were rehydrated through a series of methanol/PBST solutions in the reverse order as described above, then bleached with 10% H_2_O_2_/0.5× SSC/0.5% formamide, for 12 min at room temperature (RT). After blocking with a solution of PBST containing 10% bovine serum albumin (BSA) for 1 h at RT, the larvae were incubated with Alexafluor568-Streptavadin in the dark for 2 h at RT, then washed 3 × with PBST and stored in 80% glycerol at 4 °C.

For imaging, 5 fixed and stained larvae were mounted in 2% low melt agarose on coverslips affixed to the bottom of 3.5 mm plastic Petri dishes, on their left side such that the liver was oriented toward the objective of an inverted microscope.

### Microscope configuration and image acquisition

Images were acquired using a CSU-W1 spinning-disk confocal (Yokogawa, Japan) with 25 µm Disk, Aperture of 10 and speed 4000 rpm, mounted on an inverted Nikon Ti-Eclipse (Nikon Instruments Inc, Japan) equipped with a Nikon Plan Apo 10×/0.45NA objective lens (Nikon Instruments Inc, Japan).

Alexafluor568 fluorescence was excited with the 561 nm laser line from a multi–Laser Unit (LUN-F) and collected by using the ET605/52 band pass emission filter (Chroma Technology, USA) and the DM405/488/532/647 dichroic mirror (Yokogawa, Japan).

Images were acquired with an Andor Zyla (VSC-03133, Andor Technology, UK) controlled with NIS-Elements (v. AR 5.41.02, 64-bit, Nikon Instruments Inc, Japan) software, with binning 1 × 1, at resolution of 2048 × 2048 pixels, in 16-bit, dual Gain 1/4, and saved in Nd2 file format.

Z-stack images of the liver were collected with a step size of 2.5 µm with the Motorized Scanning Stage (Nikon Instruments Inc, Japan) and the Ti ZDrive Device. The Z-stack range was manually adjusted for each sample to yield the best possible detection.

### Image anonymization and analysis

Prior to image analysis in Imaris software (v.9.5., Bitplane, United Kingdom), each file was renamed with a random designation so that the analysis was performed blind to the identity of the sample. After the measurements were made the file names were decoded to identify the samples as wildtype of *klf9*^−/−^. After randomization, using Imaris, the images in *nd2* format were converted to *ims* format for segmentation and analysis. The liver was segmented manually by outlining the liver’s Alexafluor568 fluorescence signal, by navigation through the Z-stack and using the surface tool. The following sequence of commands and steps were used: (1) ‘Create a new surface’, (2) ‘Turn off the volume’, (3) check ‘skip auto creation’, (4) under ‘draw’ click ‘visibility (none)’. The segmented rendered surface was used as a mask and used to obtain the liver volume.

For statistical analysis, we combined the results of two sets of measurements made independently by different individuals. Preliminary analysis of these data revealed a slight systematic difference between the sizes measured by each analyst. We calculated the average value for each set of measurements and found that after each subset was zero-centered, the resulting distributions were statistically equivalent, and therefore they were joined into one set for comparative analysis.

## Supplementary Information

Supplementary Figures.

Supplementary Legends.

Supplementary Table S1.

Supplementary Table S2.

Supplementary Table S3.

Supplementary Table S4.

Supplementary Table S5.

Supplementary Table S6.

Supplementary Table S7.

## Data Availability

All RNA-seq data, along with count matrixes and DESeq2 output files have been deposited in NCBI’s Gene Expression Omnibus (GEO) under accession number GSE218687 and can be accessed via the following link: GEO Accession viewer (nih.gov). All other data will be made available by the corresponding author upon reasonable request.
