# Supplementary material for: Loss of Krüppel-like factor 9 deregulates both physiological gene expression and development
Source: Sci Rep. 2023 Jul 28;13:12239. doi: 10.1038/s41598-023-39453-3 (PMC10382561; doi:10.1038/s41598-023-39453-3)
Supplement: Supplementary file 1 — Supplementary Figures. [file 41598_2023_39453_MOESM1_ESM.pdf]

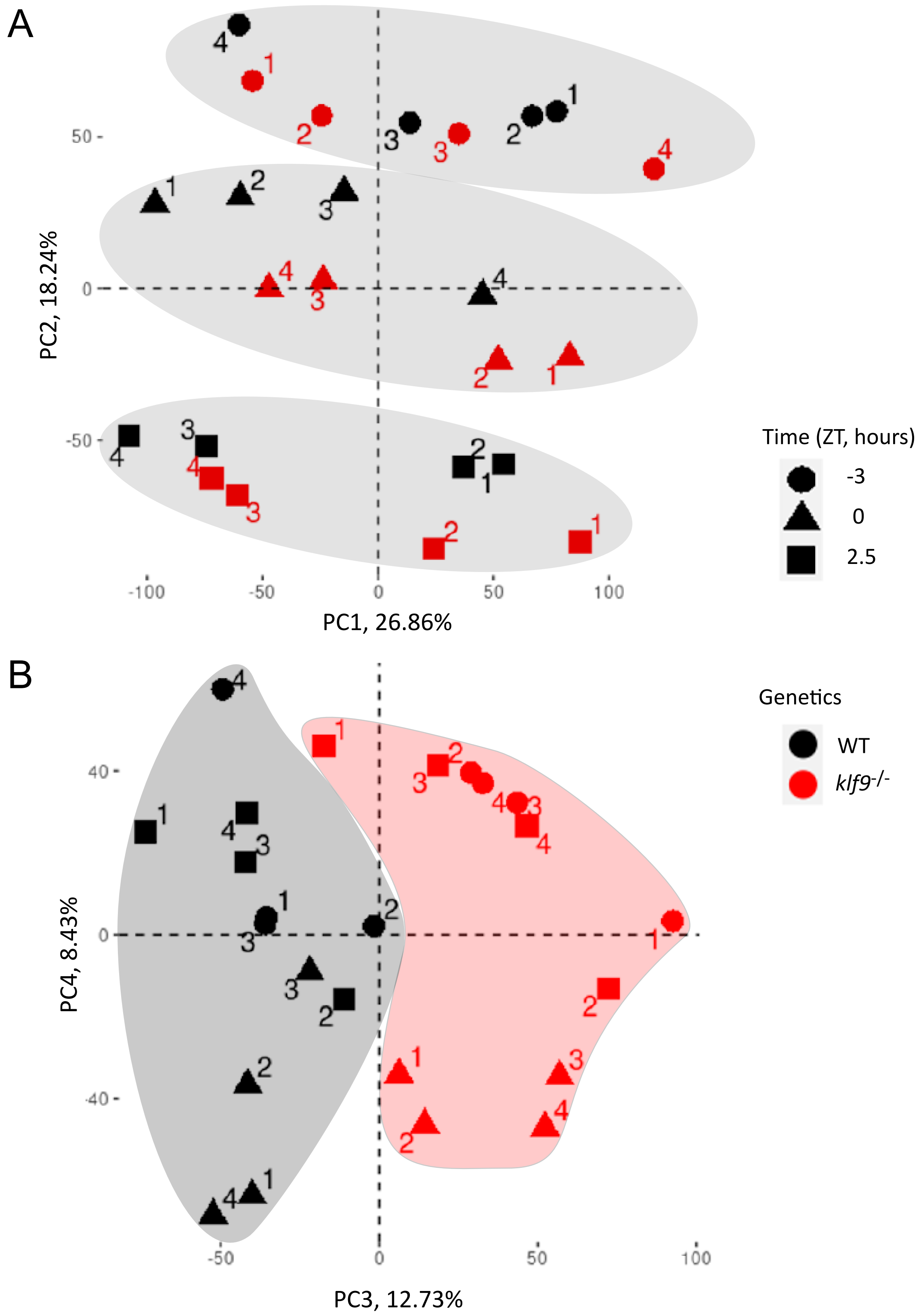

Fig. S1. Principal Component (PC) Plots of the variance among all RNA-seq samples, coded with respect to time of day (different shapes) and genotype (different colors). (A) PC1 vs PC2; (B) PC3 vs PC4.

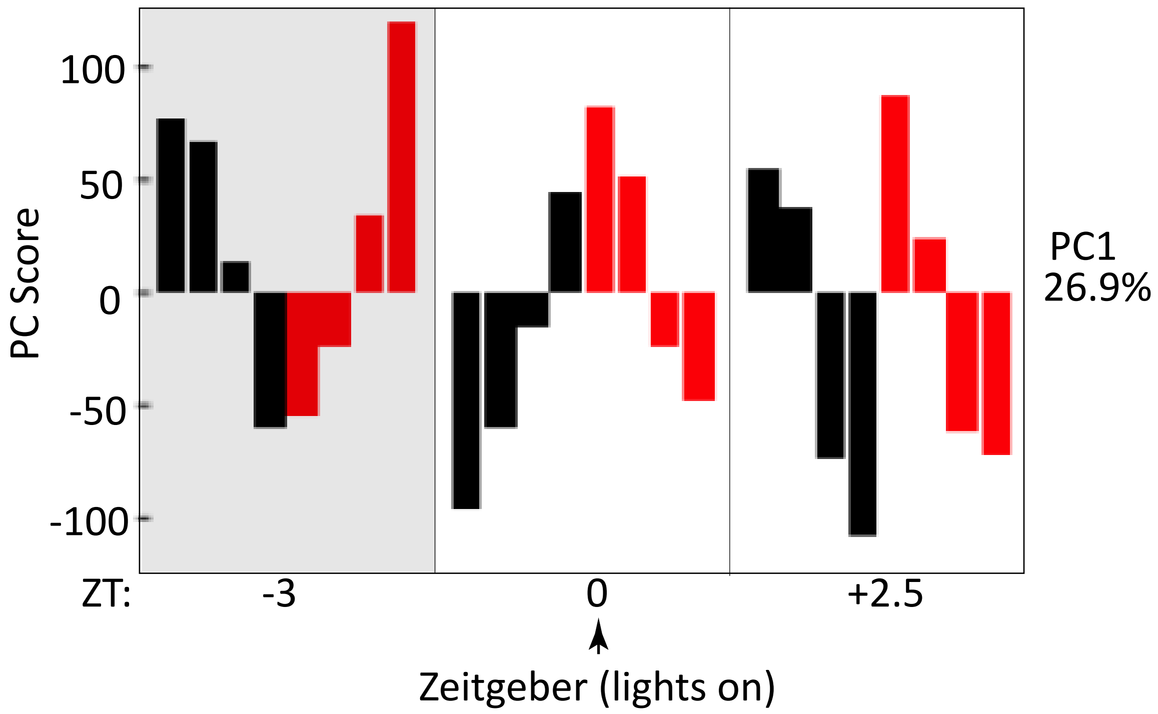

Fig. S2. Principal Component 1 captures systematic variation between biological replicates.

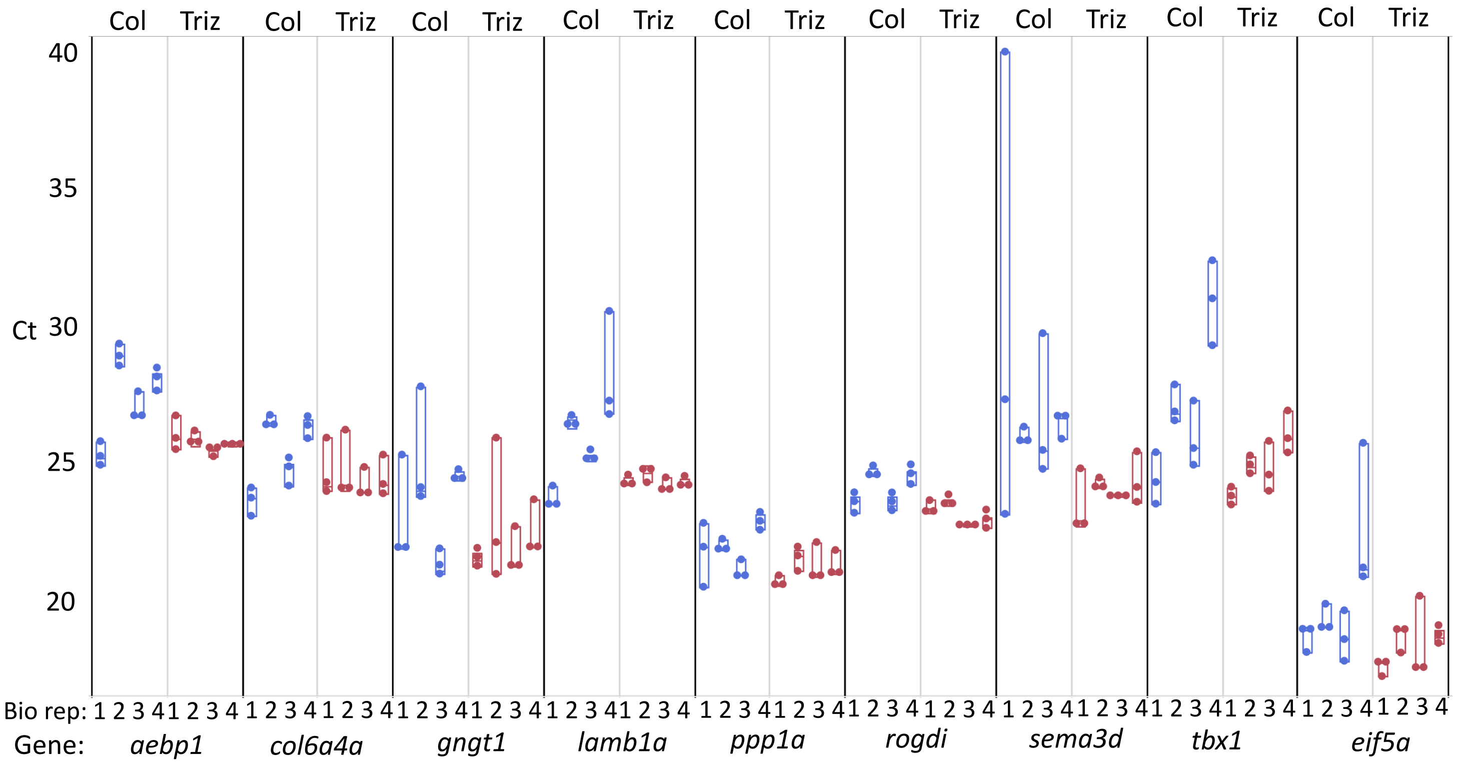

Fig S3. Use of a column kit for RNA purification without controlling for incubation time in the RLT+ buffer introduces systematic variation between replicate samples that is eliminated by use of the Trizol method and protocol that controls for incubation time. Biological replicates are on the x-axis, Cts measured for each gene on the y-axis, and each box in the plot encompasses the three technical replicates of the qPCR run.

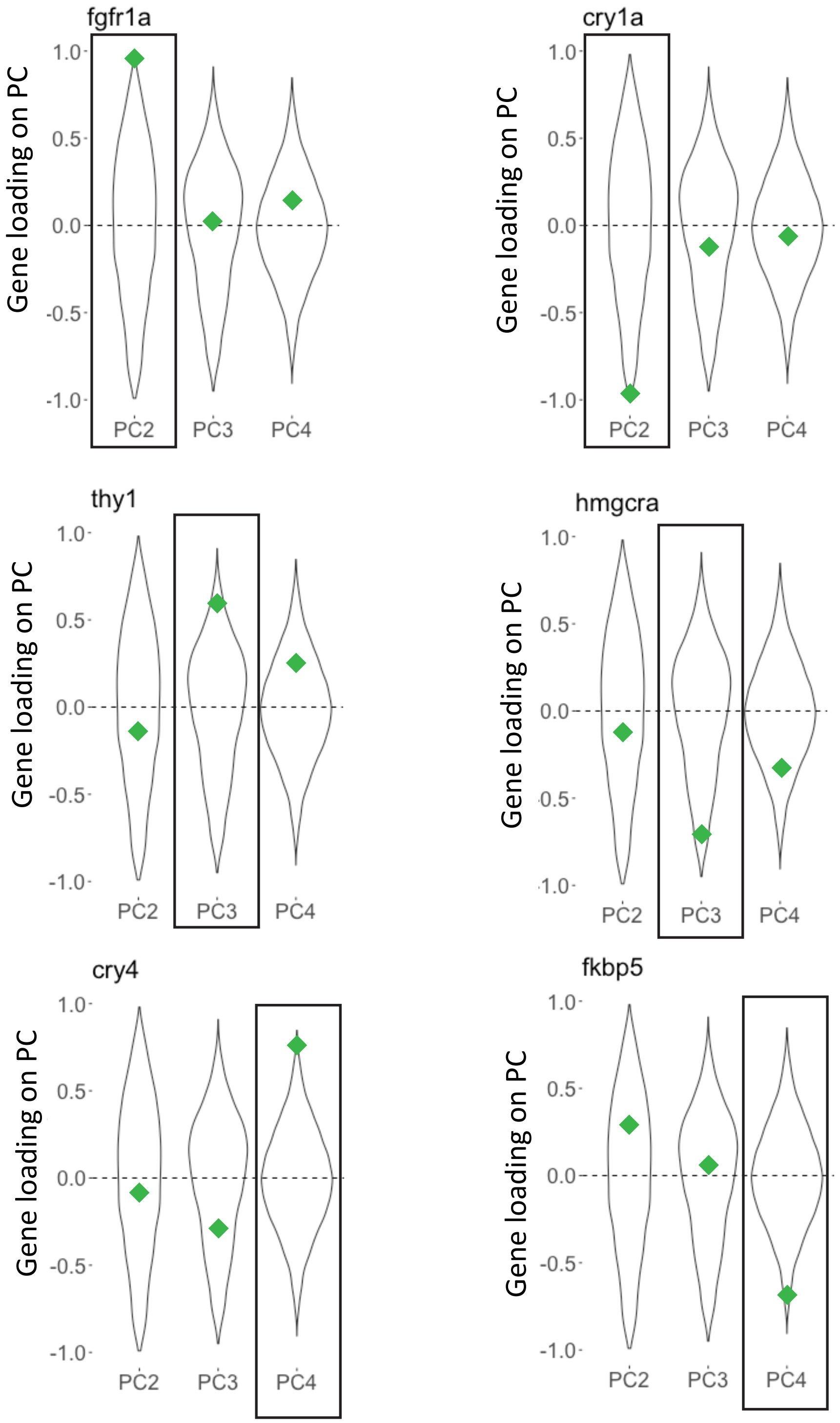

Fig. S4. Loading of genes (representing correlation between expression and PC-coordinate across all samples) shown in Figure 1 with respect to Principal Components 2, 3, and 4. The violin plots show the distribution of loading values for all genes included in the PCA.

A Gene list ranked by Principal Component 3

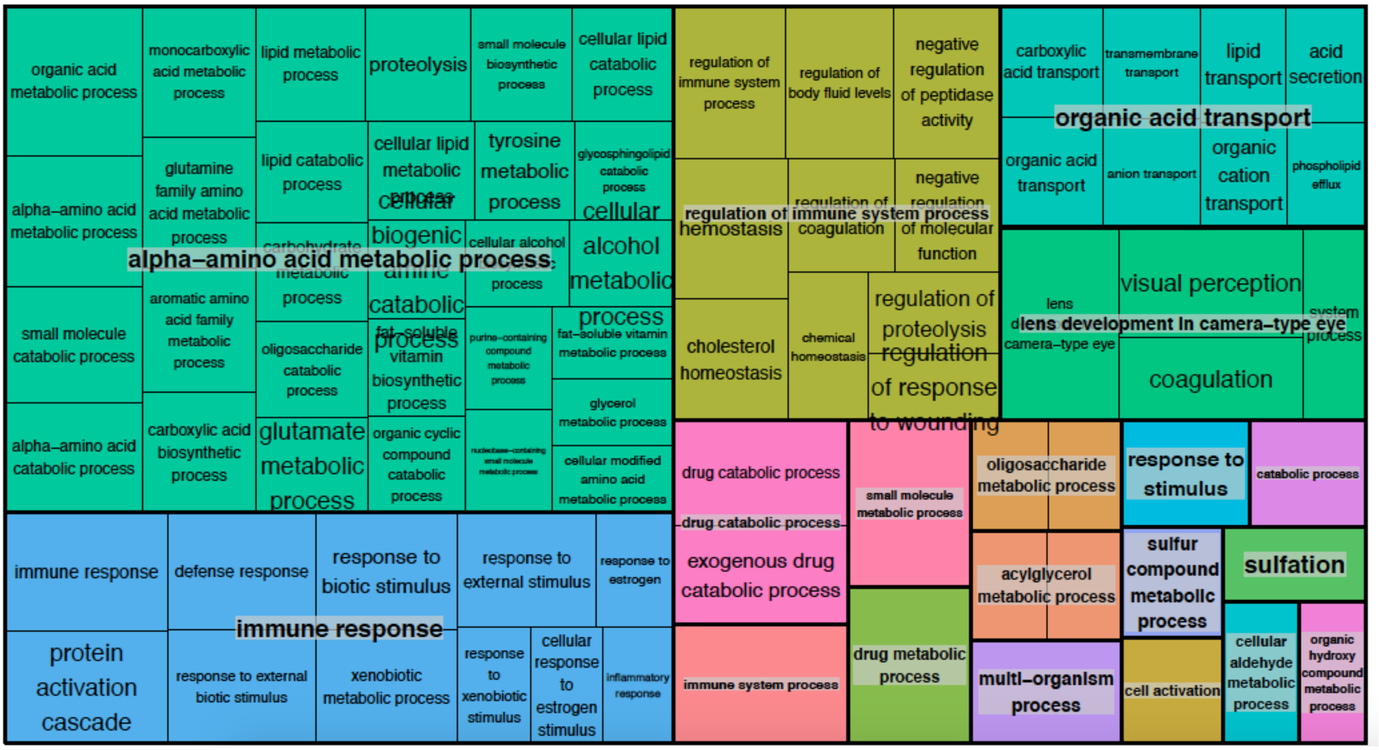

B Gene list ranked by DESeq

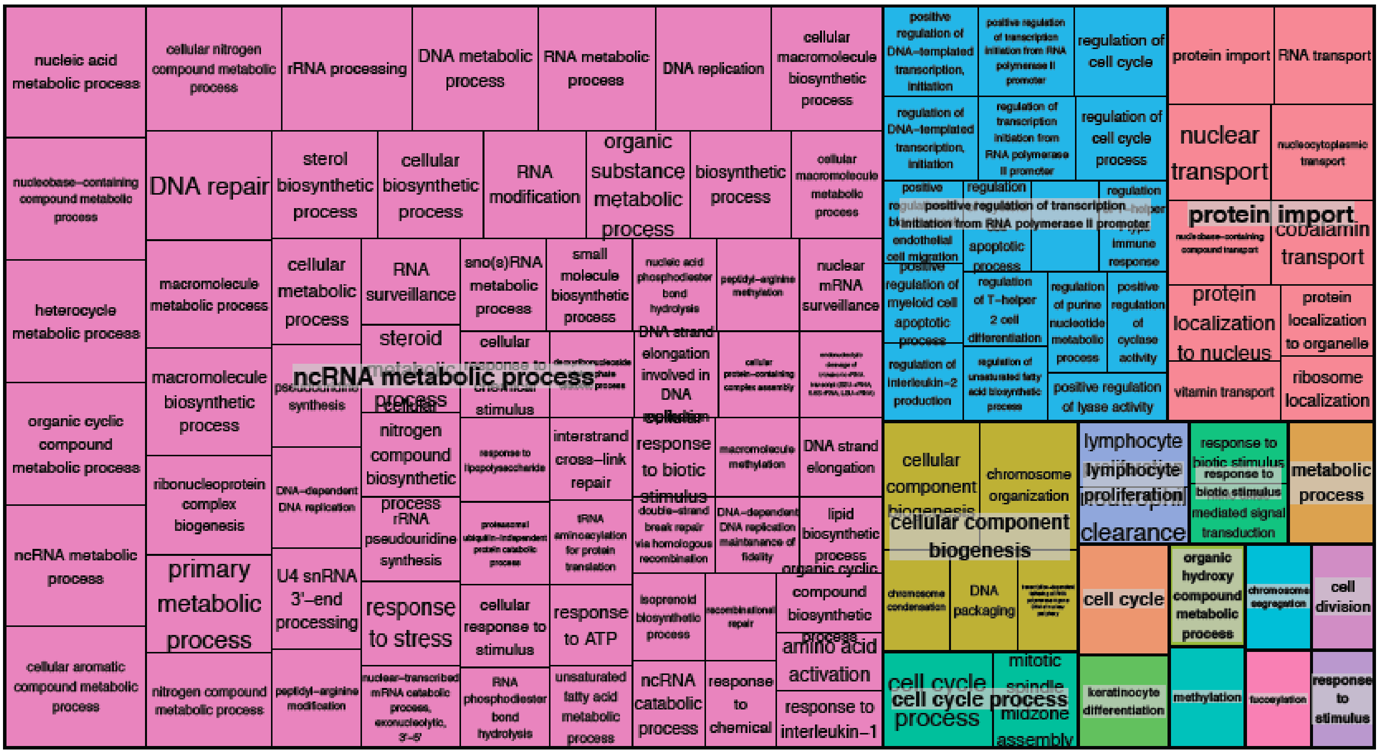

Fig. S5. Biological processes associated with genes downregulated in *klf9*$^{-/-}$ mutants. ReviGO treemaps of GOrilla Biological Process term enrichment analysis of (A) genes ranked according to their position along PC3 and (B) genes ranked according to their differential expression by DESeq.

A Gene list ranked by Principal Component 3

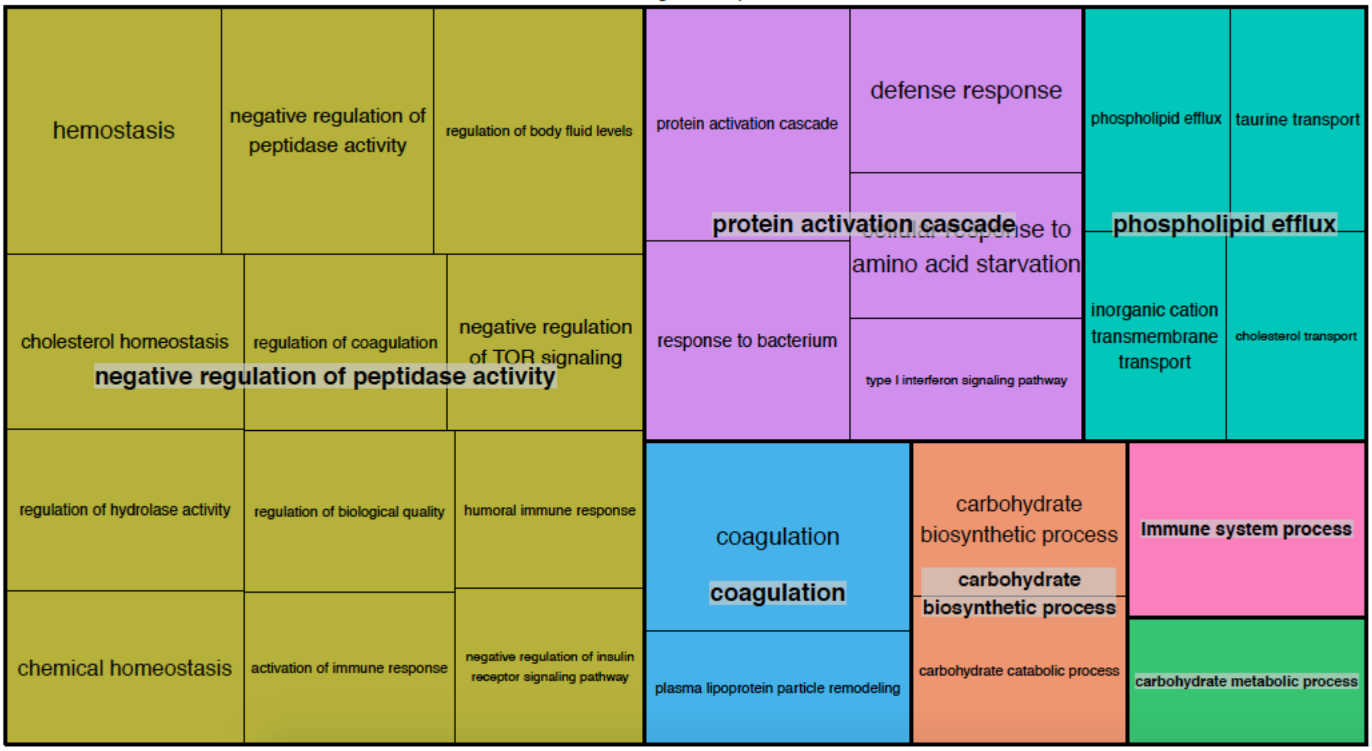

B Gene list ranked by DESeq

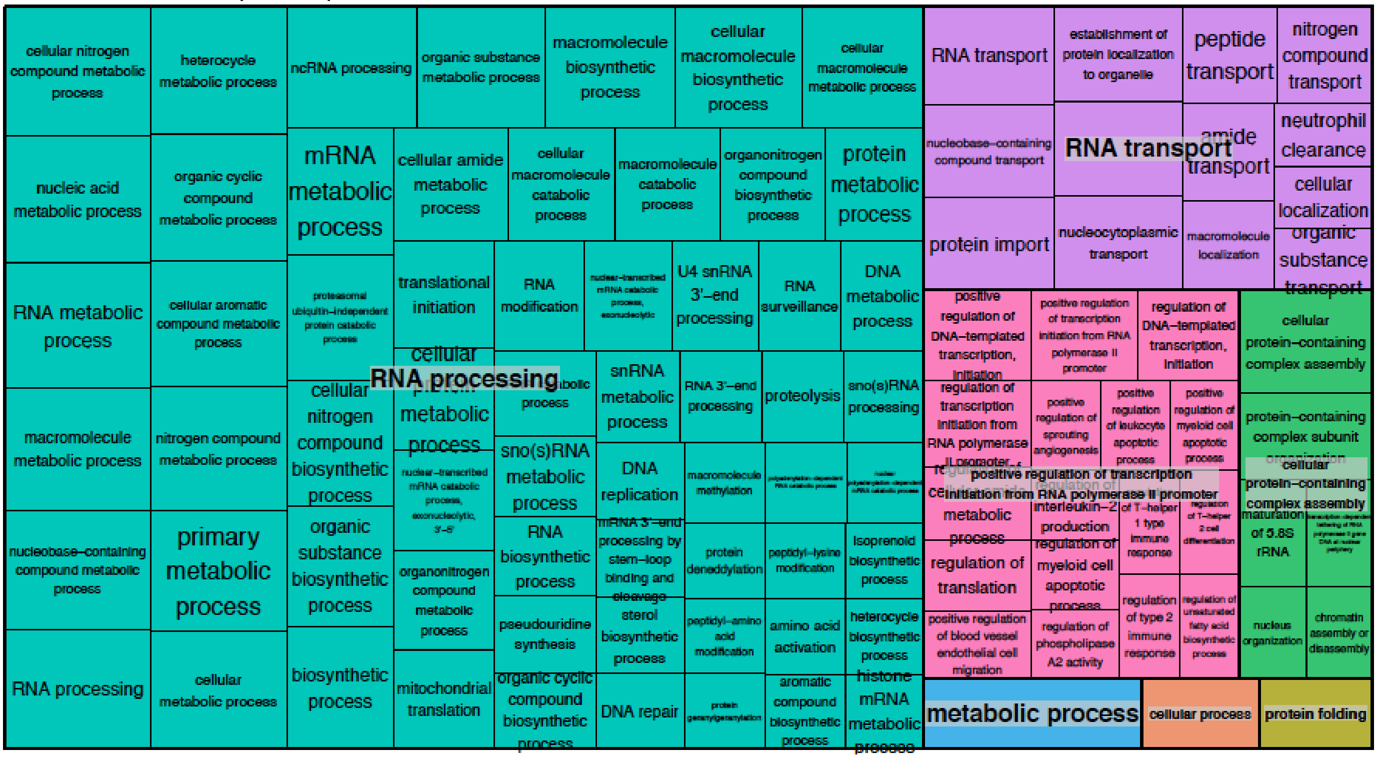

Fig. S6. Biological processes associated with genes upregulated in *klf9*$^{-/-}$ mutants. ReviGO treemaps of GOrilla Biological Process term enrichment analysis of (A) genes ranked according to their position along PC3 and (B) genes ranked according to their differential expression by DESeq.

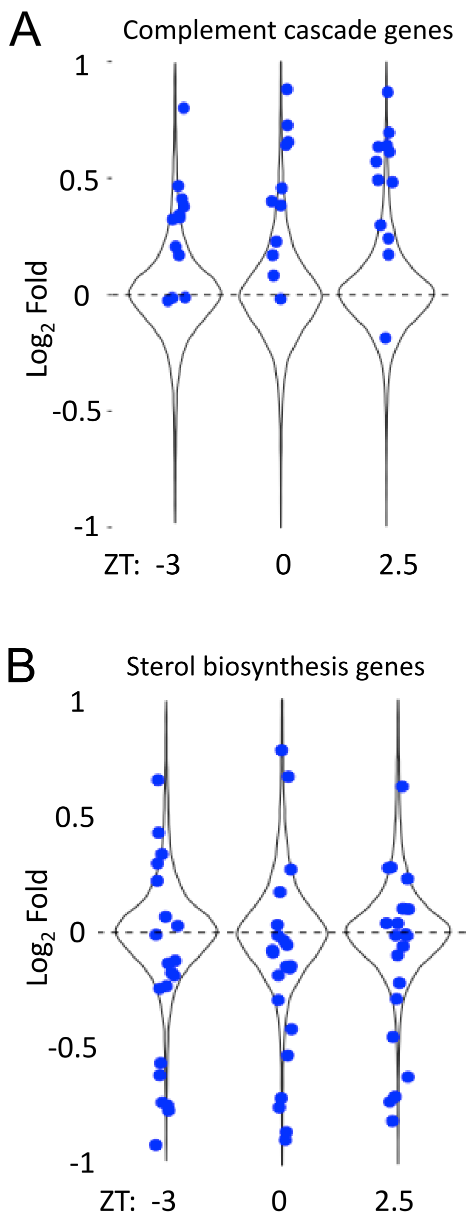

Fig. S7. Genes associated with the (A) complement system and (B) sterol biosynthesis are differentially expressed in *klf9*$^{-/-}$ mutants irrespective of time of day. The violin plots show the distribution of log2foldchange values for all genes included in the analysis (genes listed in Supplementary Table S7).

A Boiological processes associated with genes upregulated in *klf9*-/- mutants at ZT-3

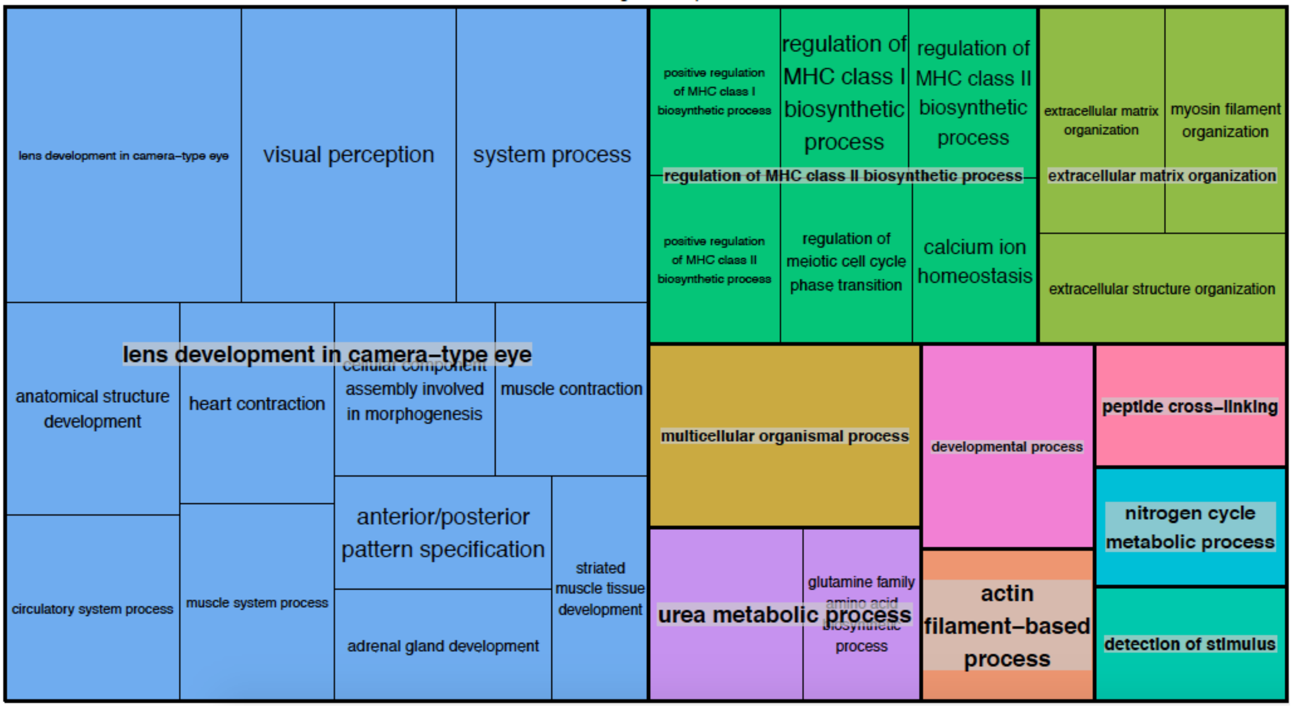

B Boiological processes associated with genes downregulated in *klf9*-/- mutants at ZT-3

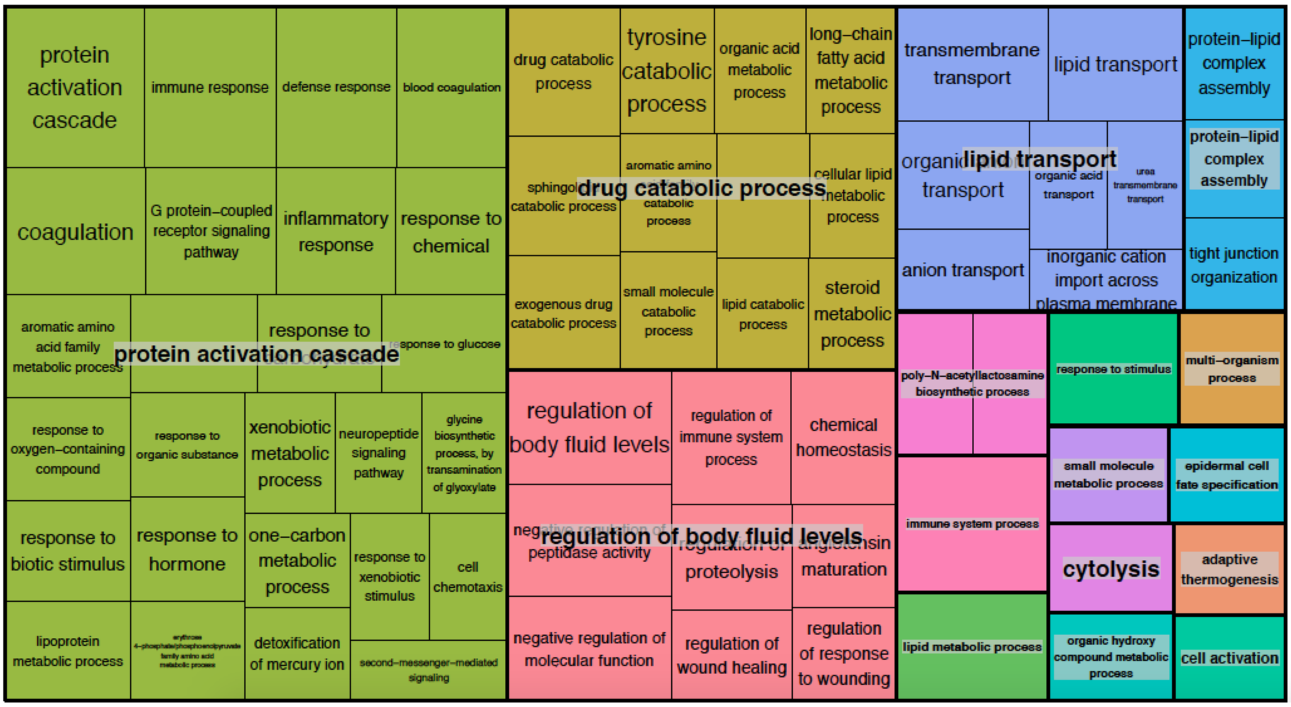

Fig. S8. Biological processes associated with genes differentially expressed in *klf9*$^{-/-}$ mutants at ZT-3. ReviGO treemaps of GOrilla Biological Process term enrichment analysis of (A) upregulated genes and (B) downregulated genes.

A Boiological processes associated with genes upregulated in *klf9*-/- mutants at ZT0

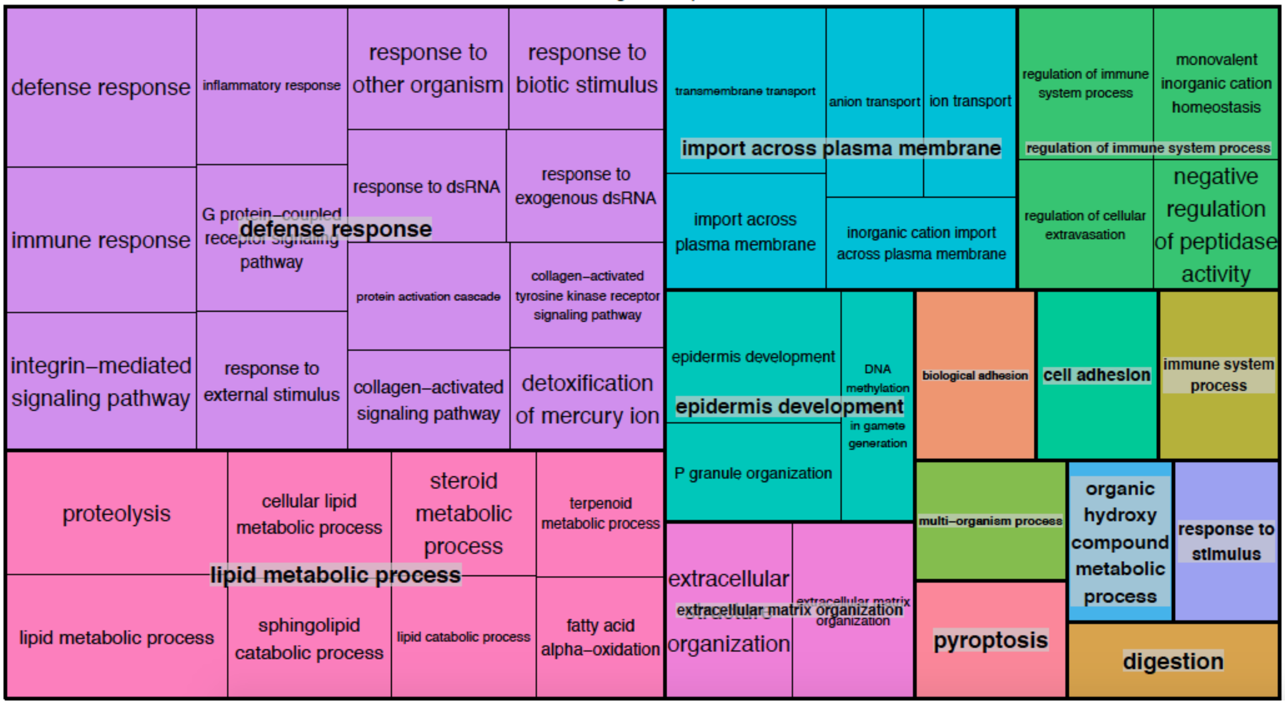

B Boiological processes associated with genes downregulated in *klf9*-/- mutants at ZT0

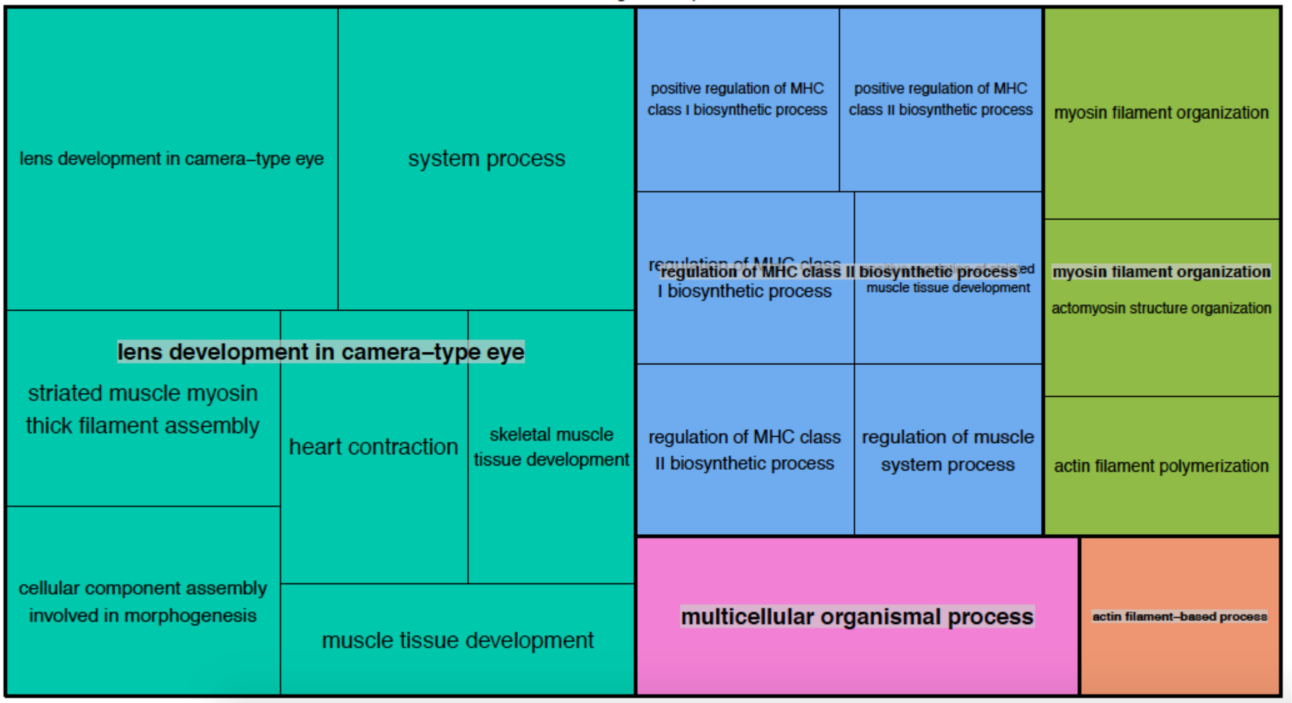

Fig. S9. Biological processes associated with genes differentially expressed in *klf9*$^{-/-}$ mutants at ZT0. ReviGO treemaps of GOrilla Biological Process term enrichment analysis of (A) upregulated genes and (B) downregulated genes.

A Boiological processes associated with genes upregulated in *klf9*-/- mutants at ZT+2.5

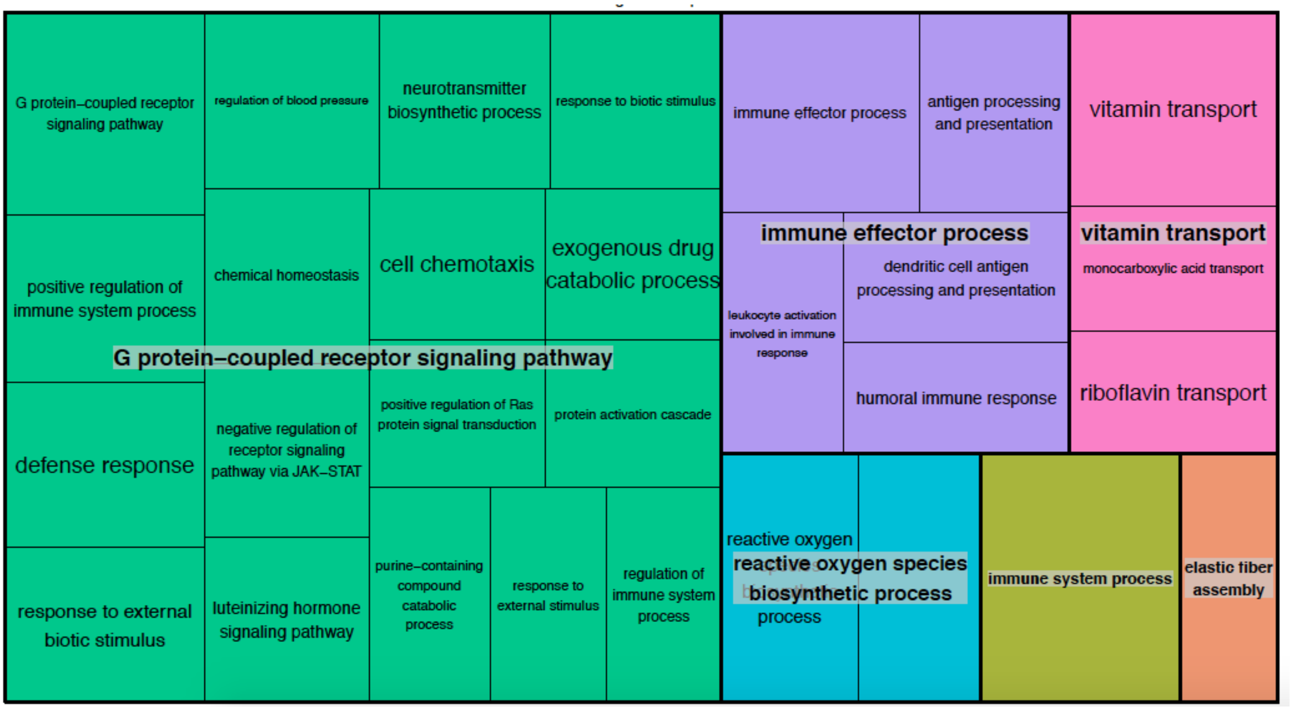

B Boiological processes associated with genes downregulated in *klf9*-/- mutants at ZT+2.5

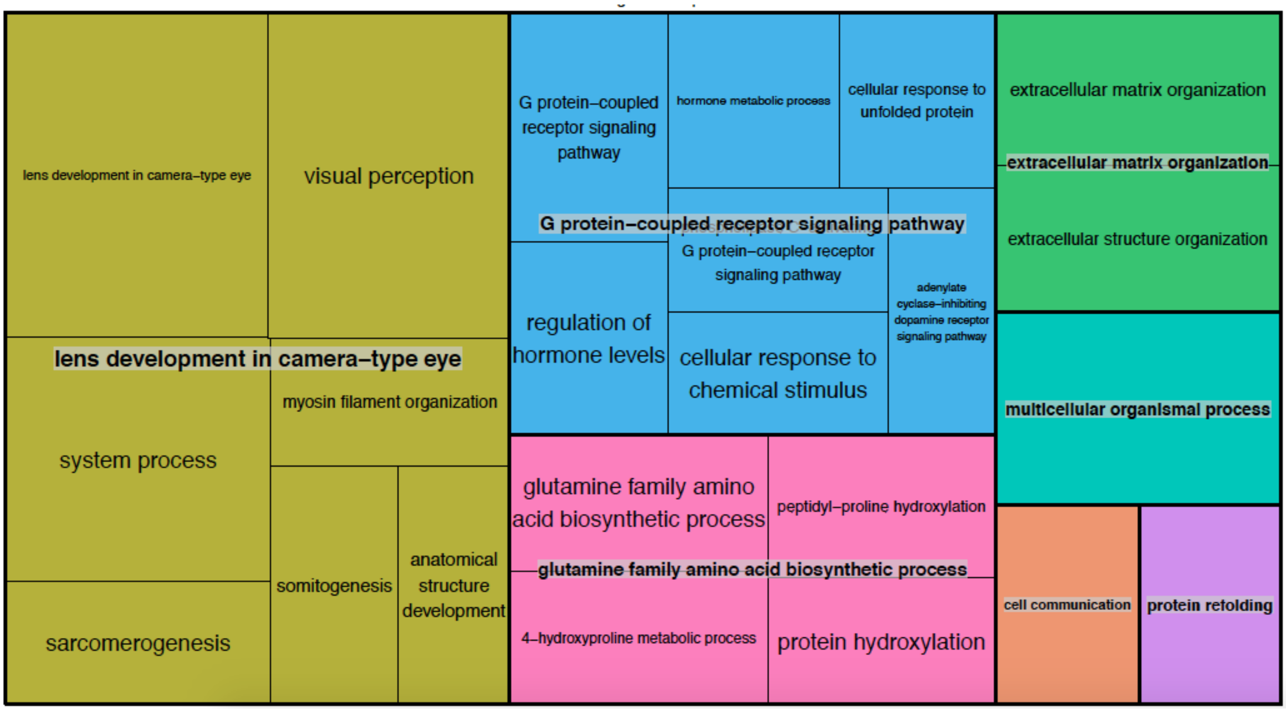

Fig. S10. Biological processes associated with genes differentially expressed in *klf9*$^{-/-}$ mutants at ZT+2.5. ReviGO treemaps of GOrilla Biological Process term enrichment analysis of (A) upregulated genes and (B) downregulated genes.

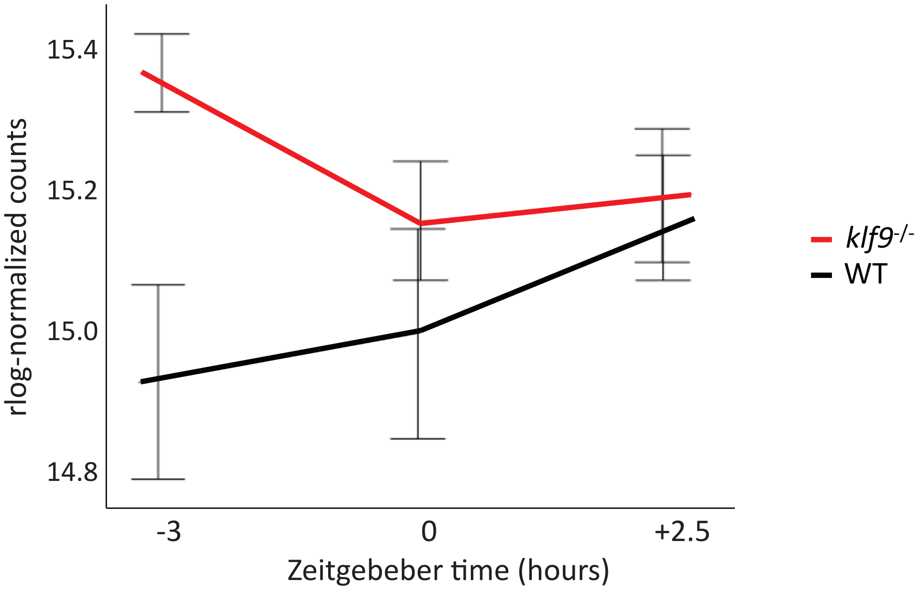

Fig. S11. Temporal profile of *cryba1b* expression. Error bars are the SEM of the four biological replicates.

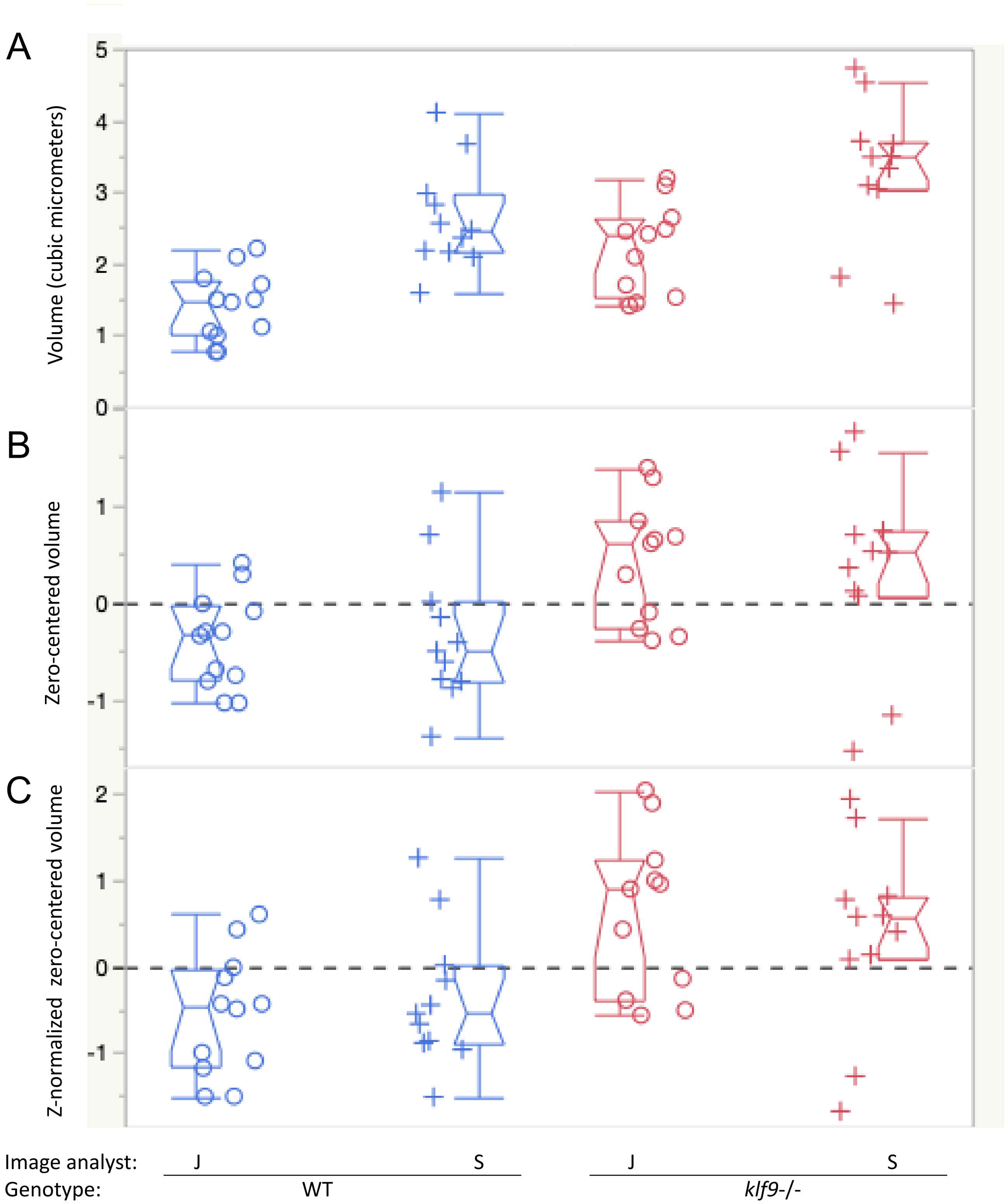

Fig. S12. Liver volume measurements plotted with respect to genotype (WT vs. *klf9*$^{-/-}$) and image analyst (J vs. S). (A) Volumes as initially calculated from the images; (B) Mean-centered volumes calculated by subtracting the average volume obtained for all the sample images by each analyst from each measurement; (C) Z-normalization of the zero-centered volumes.
