# Supplementary material for: Loss of Krüppel-like factor 9 deregulates both physiological gene expression and development
Source: Sci Rep. 2023 Jul 28;13:12239. doi: 10.1038/s41598-023-39453-3 (PMC10382561; doi:10.1038/s41598-023-39453-3)
Supplement: Supplementary file 2 — Supplementary Legends. [file 41598_2023_39453_MOESM2_ESM.docx]

**Loss of Krüppel-like factor 9 deregulates both physiological gene expression and development**

Laura Drepanos^3^, Ian M. Gans^1,2^, Janelle Grendler^1^, Sophia Guitar^1^, J. Heath Fuqua^1^, Nathaniel J. Maki^1^, Andrea R. Tilden^3^, Joel H. Graber^1^, and James A. Coffman^1,2^*

^1^MDI Biological Laboratory, Salisbury Cove, Maine, USA

^2^Graduate School of Biomedical Sciences and Engineering, University of Maine, Orono, Maine, USA

^3^Colby College, Waterville, Maine, USA

*Correspondence: [jcoffman@mdibl.org](mailto:jcoffman@mdibl.org)

**List of Supplementary Tables**

**Table S1. Nanostring Probes.** The probe sequences used to detect the genes described in the text are denoted with asterisks.

**Table S2. PCR Primers.** The table lists the sequences of the PCR primers used to test the effects of different methods of RNA purification.

**Table S3. Annotation of cell clusters from the single cell atlas of Farnsworth et al. (2020; reference 26).** The first tab lists the full table of genes, and the second lists those which passed the specificity threshold criteria of adjusted p-value <= 0.05 and pct.1 >= 0.4.

**Table S4. Correlation of expression variance of each gene with each Principal Component.** The table lists the Pearson correlation between the expression variance of each gene and each of the first four Principal Components.

**Table S5. DESeq2 results comparing gene expression in wildtype and klf9-/- larvae.** The four tabs list the DESeq2 results for all three time points combined and for each of the three time points individually.

**Table S6. Venny analysis results, showing the identities of the genes enumerated in Fig. 2a.**

**Table S7. Identities and DESeq2 results for the complement and sterol biosynthesis genes shown in Supplementary Figure S7.**
